# Assigning Quantitative Function to Post-Translational Modifications Reveals Multiple Sites of Phosphorylation That Tune Yeast Pheromone Signaling Output

**DOI:** 10.1371/journal.pone.0056544

**Published:** 2013-03-12

**Authors:** David Pincus, Christopher J. Ryan, Richard D. Smith, Roger Brent, Orna Resnekov

**Affiliations:** 1 Molecular Sciences Institute, Berkeley, California, United States of America; 2 Pacific Northwest National Laboratory, Richland, Washington, United States of America; Centre National de la Recherche Scientifique, France

## Abstract

Cell signaling systems transmit information by post-translationally modifying signaling proteins, often via phosphorylation. While thousands of sites of phosphorylation have been identified in proteomic studies, the vast majority of sites have no known function. Assigning functional roles to the catalog of uncharacterized phosphorylation sites is a key research challenge. Here we present a general approach to address this challenge and apply it to a prototypical signaling pathway, the pheromone response pathway in *Saccharomyces cerevisiae*. The pheromone pathway includes a mitogen activated protein kinase (MAPK) cascade activated by a G-protein coupled receptor (GPCR). We used published mass spectrometry-based proteomics data to identify putative sites of phosphorylation on pheromone pathway components, and we used evolutionary conservation to assign priority to a list of candidate MAPK regulatory sites. We made targeted alterations in those sites, and measured the effects of the mutations on pheromone pathway output in single cells. Our work identified six new sites that quantitatively tuned system output. We developed simple computational models to find system architectures that recapitulated the quantitative phenotypes of the mutants. Our results identify a number of putative phosphorylation events that contribute to adjust the input-output relationship of this model eukaryotic signaling system. We believe this combined approach constitutes a general means not only to reveal modification sites required to turn a pathway on and off, but also those required for more subtle quantitative effects that tune pathway output. Our results suggest that relatively small quantitative influences from individual phosphorylation events endow signaling systems with plasticity that evolution may exploit to quantitatively tailor signaling outcomes.

## Introduction

In response to stimuli sensed at the cell surface by receptors, eukaryotic cells propagate signal to the nucleus via intracellular signaling pathways. Such pathways inform decisions about cell fate, cell polarity, migration, cell-cycle regulation, cell proliferation and programmed cell death [1], [2], [3], [4], [5]. Signal transmission is often accomplished by regulated phosphorylation of protein components of signaling pathways. Phosphorylation can rapidly and reversibly modulate numerous properties of proteins including their conformation, enzymatic activity, molecular interactions, subcellular localization and surface charge [6], [7].

In organisms as diverse as mammals, invertebrates and yeast, researchers have used mass spectrometry based proteomics to identify thousands of sites of phosphorylation on proteins in cell signaling pathways. Some sites of phosphorylation serve as molecular switches that activate an enzyme (e.g., sites on the activation loop of kinases), determine a protein’s subcellular localization (e.g., sites that exclude a transcription factor from the nucleus), or target a protein for degradation (e.g., cell-cycle controlled phospho-degrons) [8], [9], [10], [11]. The roles of these types of sites are relatively straightforward to elucidate with qualitative assays since point mutants that cannot be phosphorylated either phenocopy null mutants or produce constitutive activity. However, there are many uncharacterized phosphorylation sites. We hypothesized that many of these sites exert quantitative regulatory roles in signaling pathways, the effects of which would only be revealed with quantitative assays in the context of the specific stimulus about which they convey information. We therefore developed a systematic and general approach to prioritize the study of individual phosphorylation events and their potential quantitative functions in signaling networks.

We focused on the pheromone response system in the budding yeast *Saccharomyces cerevisiae* – a well-developed system for studying eukaryotic cell signaling. In this system we can sensitively and accurately quantify pathway input, signal flow and pathway output *in vivo* in single cells [12], [13], [14].

**Figure 1:**
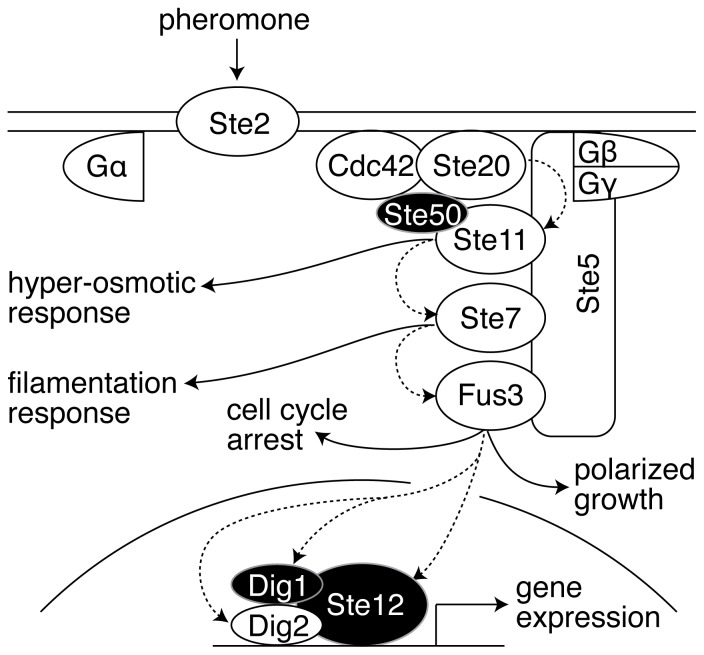
Pheromone induced mating pathway in yeast. Pheromone (α factor) binds to a G-protein coupled receptor (Ste2). Binding promotes dissociation of the heterotrimeric G protein into Gα and Gβγ. Free Gβγ initiates downstream signaling by forming a complex with the scaffold protein Ste5 at the plasma membrane, as well as the small GTPase Cdc42, the adaptor protein Ste50 and the kinase Ste20. Ste5 further recruits the MAPK cascade members, Ste11, Ste7 and Fus3. Once this complex forms, Ste20 phosphorylates and activates Ste11; Ste11 then phosphorylates and activates Ste7; Ste7 phosphorylates and activates the MAPK Fus3. Dashed arrows indicate phosphorylation of proteins involved in the pheromone response shown here; solid arrows show connections to the high osmolarity response pathway, filamentation pathway, cell cycle control and polarized growth. Ste11 also participates in the hyper-osmotic response and the filamentation MAPK pathways, and Ste7 also participates in the filamentation pathway. Active Fus3 executes the different cellular responses to pheromone by phosphorylating and activating the transcriptional complex of Ste12, Dig1 and Dig2 to express the pheromone responsive target genes. In addition to transcriptional activation, Fus3 also arrests the cell cycle and initiates polarized growth. Figure omits other phosphorylation events and feedback. Phosphorylation events on the proteins Ste50, Dig1 and Ste12 (shaded in black) are the focus of this work.

The pheromone signaling system is stimulated by binding of a 13 amino acid peptide pheromone to a 7-transmembrane spanning G protein-coupled receptor (GPCR), activating a mitogen-activated protein kinase (MAPK) cascade and triggering downstream cellular events including gene transcription, cell cycle arrest, cell fusion and mating (Figure 1) [15]. Upon GPCR activation by pheromone, the Gα subunit of the heterotrimeric G-protein binds GTP and dissociates from Gβγ. Free Gβγ diffuses in the plasma membrane and provides a binding site that recruits the scaffold protein Ste5. At the plasma membrane, Ste5 recruits members of the MAPK cascade: the MAP3K Ste11, the MAP2K Ste7, and the MAPK Fus3. To initiate signaling, Ste11 is phosphorylated by the p21-activated kinase (PAK) Ste20, which is activated by binding to the GTP-bound form of the small GTPase Cdc42. To bridge an interaction between Ste11 and active Ste20, Ste11 binds to the adaptor protein Ste50; Ste50 also binds to Cdc42, thus bringing Ste11 to active Ste20 [16], [17]. After activation by Ste20, Ste11 phosphorylates Ste7, which in turn phosphorylates Fus3 in the presence of Ste5 [18], [19]. Fus3 (an effector kinase) phosphorylates substrates in the cytoplasm to induce cell cycle arrest and polarized growth. In the nucleus, Fus3 phosphorylates the functionally redundant transcription repressors Dig1 and Dig2, as well as the transcription activator Ste12 to induce pheromone responsive gene expression [20], [21]. Upon stimulation with pheromone, precise quantitative information about the amount of receptor occupied at the cell surface reaches the nucleus within minutes [12], [13], [22].

Here, we mined mass spectrometry based proteomics data to identify phosphorylation sites with no known function on components of the pheromone pathway. We then prioritized for further examination phosphopeptides that contained conserved consensus sequences (associated with MAPKs) in regions of system proteins that contained no known or predicted structural domains. Finally we combined mutations, quantitative single-cell experiments and computational modeling to define novel quantitative roles for six phosphorylation events on three pathway proteins in regulating pheromone signaling. While more detailed and mechanistic computational models of the pathway have been published before, the simple models used here are course-grained in order to be generic and broadly applicable [23], [24], [25].

We found that mutation of three of the six sites decreases system output, mutation of two other sites increases system output, and mutation of the final site removes a negative feedback loop that conditionally diminishes system output when signal is low. We believe that a similar combination of approaches will allow researchers to characterize functional roles for phosphorylation events that contribute to the dynamic quantitative regulation of many signaling systems.

## Results

### Experimental overview: identifying putative sites of phosphorylation and assaying mutants for quantitative pheromone signaling phenotypes

Ste12, Ste50 and Dig1 function at different points in the pheromone response system upstream and downstream of the protein kinase cascade (Figure 1). We chose 4 tryptic peptides containing reported phosphorylation sites: two on Ste12 (L398-K409 and P523-R529), one on Ste50 (R200-R208), and one on Dig1 (V266-K282) (Table S4). We prioritized these peptides based on the following criteria: (1) Each peptide contains a residue predicted to be a MAP kinase substrate within a consensus MAP kinase site (S/T-P); (2) the MAPK consensus motifs are conserved from *S. cerevisae* to at least *S. bayanus* (∼20 million years); (3) the chosen peptides lie outside of evolutionarily conserved predicted structural domains (STE on Ste12; SAM and RA domains on Ste50) [26], [27], [28], [29], [30].

We made individual haploid yeast strains in which we replaced wild type *STE12, STE50* or *DIG1* with a mutant allele encoding the protein to be tested under the control of its native promoter at its endogenous locus (Table S1, Table S2, Table S3). We mutated single serine and/or threonine (S/T) residues as well as all S/T residues within the regions corresponding to the observed tryptic phosphopeptides to account for potential ambiguities in identified phosphorylation sites and the possibility of multiple phosphorylated forms. To quantitatively assess pathway output in strains containing the mutated proteins, we inserted a transcriptional reporter containing the pheromone responsive P_PRM1_ promoter fused to a fluorescent protein (either YFP or mCherry), and used previously described fluorescence microscopy and flow cytometry-based single cell assays to measure fluorescence after treatment with different concentrations of pheromone [12], [13], [31].

### Putative phosphorylation sites S400 and T525 on Ste12 are required for full induction of the pheromone response

**Figure 2:**
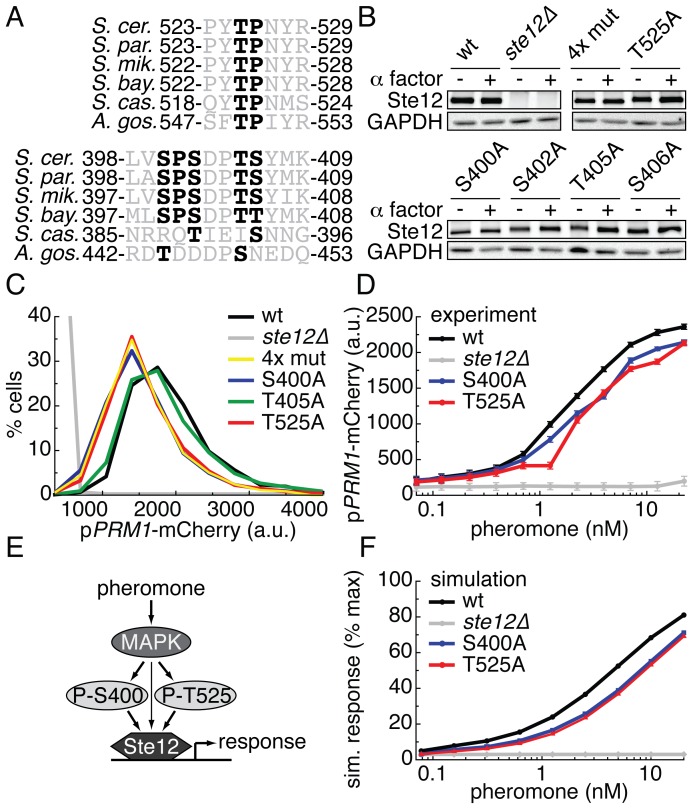
Two phosphorylation sites in Ste12 are required for full transcriptional induction. A. Alignment of 2 tryptic phospho-peptides across 6 yeast species. Possible phosphorylation sites (serines and threonines) and minimal MAPK consensus sequences (S/T,P) are in bold. B. Western blots of lysate from yeast strains containing the indicated wild type or mutant Ste12, treated or not treated with 1 µM alpha factor for 15 minutes probed with anti-Ste12 and anti-GAPDH. 4x mut is Ste12^S400A,S402A,T405A,S406A^. C. Histograms of the single cell (n > 2000 cells) pheromone response measured by p*PRM1*-mCherry fluorescence. Cells were treated with 4 nM alpha factor for 3 hrs followed by cycloheximide for 2 hrs, and measured by flow cytometry. D. Dose responses of cells treated with 12 concentrations of pheromone as described in C. Figure plots means of the unimodal distributions. Error bars depict the standard error of the mean. E. Cartoon model of the function of the phosphorylation of S400 and T525 F. Simulation of the ODE model across a dose response of pheromone.

To begin to elucidate the quantitative role of phosphorylation in the pheromone pathway, we focused on two tryptic peptides on the transcriptional activator Ste12 (Figure 2A, L398-K409 and P523-R529). Both peptides contained conserved candidate MAPK phosphorylation sites (S400 and T525) with no known roles in pheromone signaling. Phosphorylation of S400 has previously reported by several groups, and was shown to increase in abundance in cells treated with 2 µM pheromone for 2 hours, but has no known function (Table S4) [32], [33], [34], [35]. Since there were 4 potential phosphorylation sites on peptide L398-K409, we changed S400, S402, T405 and S406 to alanine individually, as well as in combination (4X mutant) (Figure 2A and B). In peptide P523-R529, we changed T525 to alanine (Figure 2A and B). All mutant versions of Ste12 were detectable by immunoblot at comparable levels before and after stimulation with pheromone (Figure 2B) [36].

To quantify pheromone pathway output in these strains we used flow cytometry to measure signal from a fluorescent reporter gene fused to a pheromone responsive promoter (P*_PRM1_-*mCherry) [12], [13], [37]. After treatment with 4 nM α-factor (pheromone), the 4X mutant strain, the Ste12^S400A^ strain, and the Ste12^T525A^ strain all displayed statistically significant ∼25% decreases in fluorescence compared to the wild type reference strain (Figure 2C, Table S5). The fluorescence in strains bearing Ste12^S402A^, Ste12^T405A^, and Ste12^S406A^ was indistinguishable from the wild type reference strain (Figure 2C, Figure S1). The diminished output of the Ste12^S400A^ and Ste12^T525A^ strains persisted across a wide range of α-factor doses (Figure 2D). These results are consistent with the notion that phosphorylation of S400 and T525 is required to fully activate Ste12.

### A simple computational model of phospho-regulation of Ste12 S400 and T525 recapitulates the quantitative mutant phenotypes

To explore how phosphorylation of S400 and T525 regulate pheromone signaling, we developed a simple mathematical model of the pheromone pathway (Figure 2E, Figure S2, Figure S3). This model omitted many of the interactions and molecular states involved in pheromone signaling, and thus minimized the number of unmeasured parameters that are commonly needed to construct chemical reaction models of biological systems [38]. Our mass action based model consisted of a set of six differential equations. Each differential equation described the rate of change in activity of a single species, and each species has parameters associated with it that describe basal synthesis rate, degradation rate, and the strength of its activity. Structurally, the models represented phospho-S400 and phospho-T525 as distinct species, and activation of Ste12 depended on their “concentration” (Figure S2). We simulated site mutants by setting the concentration of the phosphorylated species to 0 while keeping other parameter values constant. This approach facilitated simulation and exploration of different possible regulatory architectures in both reference and site mutant strains.

We based the model on two assumptions. First, since in the Ste12 sequence both S400 and T525 precede proline residues (the minimum consensus requirement for MAPK substrates), we assumed that the MAPK Fus3 phosphorylates these residues [39], [40]. Second, we assumed that phosphorylation of S400 and T525 and their effects on Ste12 were independent of each other. To explore the effects of the site mutants, we simulated both “wild type” and “mutant” models across the broad range of pheromone inputs for which we had experimental data (Figure 2F). We selected generic parameter sets and analyzed the sensitivity of the model to each parameter (Figure S3). The simulated reference and mutant circuits recapitulated experimentally observed diminution of pathway output across a broad range of parameters. Thus, the data are consistent with a model in which phosphorylation of S400 and T525 increase the gain of the system.

### Ste50 S202 inhibits signaling at low concentrations of pheromone

**Figure 3:**
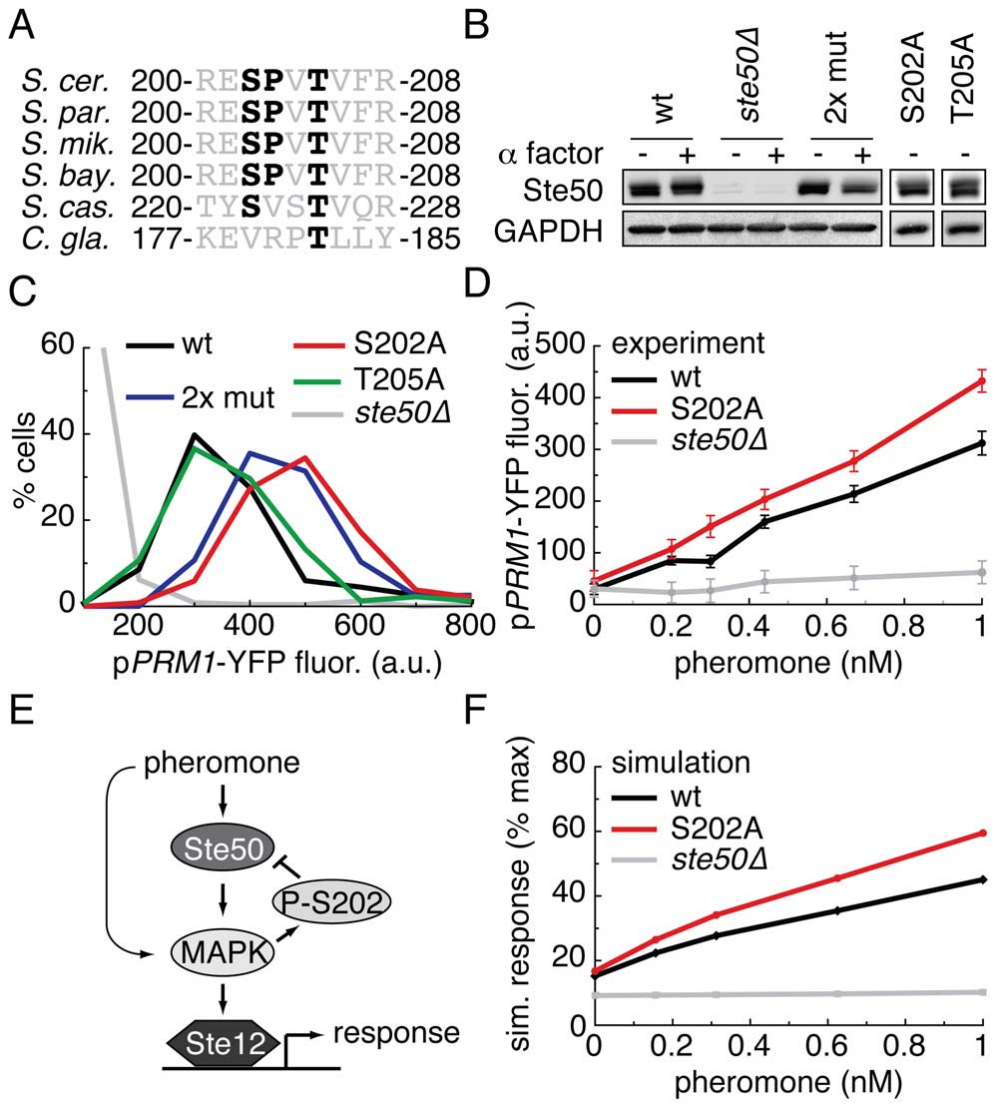
Phosphorylation of S202 on Ste50 inhibits pathway activity at low doses of pheromone. A. Alignment of a Ste50 tryptic phospho-peptide across 6 yeast species. Possible phosphorylation sites (serines and threonines) and minimal MAPK consensus sequences (S/T,P) are in bold. This peptide is not within either the SAM or the RA domains. B. Western blots of lysate from yeast strains containing the indicated wild type or mutant Ste50 that had been in the absence or presence of 1 µM alpha factor for 15 minutes probed with anti-Ste50 and anti-GAPDH. C. Histograms of the single cell pheromone response measured by p*PRM1*-YFP fluorescence. Cells (n > 300 cells) were treated with 1 nM alpha factor for 3 hrs followed by cycloheximide for 2 hrs, imaged by fluorescent microscopy, and quantified with Cell-ID. D. Dose responses of cells treated with 5 concentrations of pheromone as described in C. The means of the unimodal distributions are plotted. Error bars depict the standard error of the mean. E. Cartoon model of the function of the phosphorylation of S202. F. Simulation of the ODE model across a dose response of pheromone.

We next extended our interrogation of the quantitative phospho-regulation of the pheromone pathway to the adaptor protein Ste50 that lies upstream of the MAPK cascade. We focused our attention on the phosphorylated tryptic peptide R200-R208 (Table S4) that contains a conserved putative MAPK phosphorylation site at S202 with no known role in pheromone signaling (Figure 3A) [32], [33], [34]. We changed S202 and T205 to alanine individually, as well as in combination (2X mutant) (Figure 3A and B). Mutant versions of Ste50 were detectable by immunoblot at comparable levels (Figure 3B) [36].

To quantify pheromone pathway output in strains containing the mutated proteins, we used microscopy coupled to the Cell-ID image analysis software to measure fluorescence from P*_PRM1_-*YFP [12], [13], [41]. The strain expressing Ste50^S202A^ and the 2X mutant strain showed increased fluorescence at a low dose (1 nM) of pheromone compared to the wild type reference, while the Ste50^T205A^ strain was indistinguishable from wild type (Figure 3C). No phenotype was observed at higher doses of pheromone. To better resolve system output at low pheromone inputs we used strains that carried a mutant cyclin-dependent kinase (Cdc28-as2) sensitive to the chemical inhibitor 1-NM-PP1 [42]. In these cells, addition of 1-NM-PP1 arrests progression through the cell cycle, thus removing a major contribution of cell-to-cell variation in pheromone system output [12]. After treatment with 1-NM-PP1, Ste50^S202A^ and the 2X mutant displayed increased output when stimulated with pheromone across a dose range of 0.2–1 nM compared to the wild type reference and the Ste50^T205A^ strain (Figure 3D and Figure S4). These data suggest that phosphorylation of S202 is required to prevent hyper-activation of the pathway at low doses of pheromone.

### Computational modeling supports a negative feedback mechanism via phosphorylation of Ste50 S202

To formalize our notion that phospho-S202 prevents hyper-activation of the pheromone pathway we constructed a mathematical model. Based on the facts that Ste50 acts upstream of the MAPK cascade and S202 is a predicted MAPK substrate, we structured the model such that phospho-S202 acts as a negative feedback loop (Figure 3E, Figure S5). The model consists of five differential equations describing the rates of change of the species, including phospho-S202. Simulation of the model across the experimental pheromone dose response range recapitulated the sensitization in the S202A mutant over a wide range of parameter values (Figure 3F and S5). Our results are consistent with the idea that phosphorylation of S202 on Ste50 defines a negative feedback loop that dampens pheromone pathway output and prevents hyper-activation of the pathway at low doses of pheromone.

### Dig1 is a co-activator of transcription in the presence of CFP-Ste12

**Figure 4:**
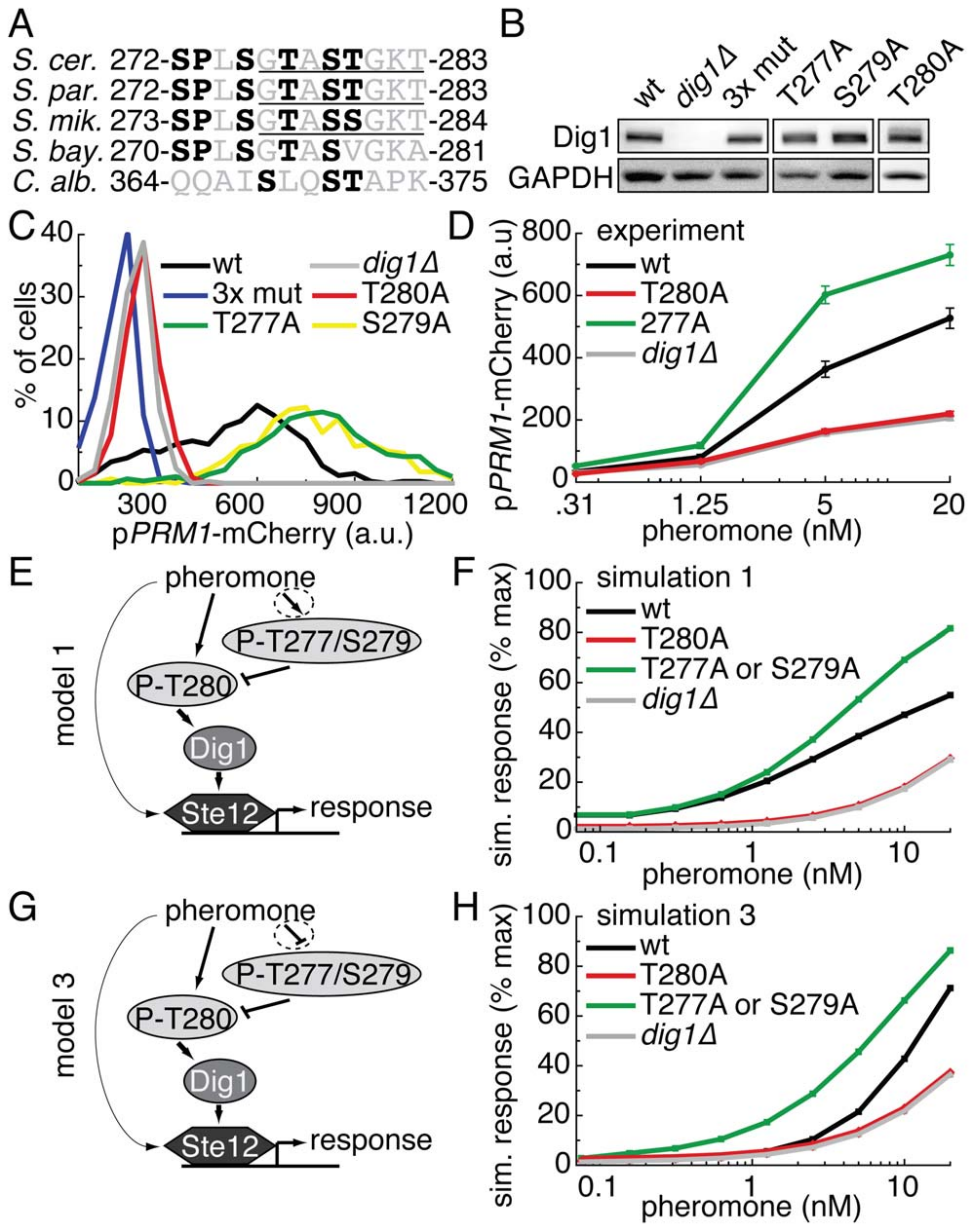
Opposing phenotypes in Dig1 phospho-mutants. A. Alignment of an 12-amino acid sequence, part of a tryptic phospho-peptide, in 5 yeast species. Possible phosphorylation sites (serines and threonines) and minimal MAPK consensus sequences (S/T,P) are in bold. The “TAST” sequence in *S. cer.* Dig1 falls in the variable region of a bioinformatically predicted ATP/GTP binding motif (p-loop, underlined). B. Western blots of lysate from yeast strains containing the indicated alleles of Dig1-YFP probed with rabbit anti-Dig1 and anti-GAPDH. 3xmut is Dig1^T277A,S279A,T280A^. C. Histograms of the single cell pheromone response measured by p*PRM1*-mCherry fluorescence. Cells (n > 500 cells) were treated with 20nM alpha factor for 3 hrs followed by cycloheximide for 2 hrs, imaged by fluorescent microscopy, and quantified with Cell-ID. D. Dose responses of cells treated with the indicated concentrations of pheromone as described in C. The means of the unimodal distributions are plotted. Error bars depict the standard error of the mean. E. Cartoon of model 1, in which pheromone induces phosphorylation of all residues. F. Simulation of model 1 across a dose response of pheromone. G. Cartoon of model 3, in which pheromone induces the dephosphorylation of T277/S279 and the phosphorylation of T280. H. Simulation of model 3 across a dose response of pheromone.

We concluded our initial examination of the quantitative phospho-regulation of the pheromone pathway by focusing on Dig1, a functionally redundant transcriptional repressor of Ste12 [43]. Though Dig1 is phosphorylated at ≥18 sites, no site has a known function [32], [33], [34], [35]. We focused on the multiply phosphorylated tryptic peptide V266-K282. V266-K282 contains a putative MAPK phosphorylation site at S272 and a predicted nucleotide-binding motif (Figure 4A).

Functionally redundant proteins present a distinct challenge to studying the functional consequences of individual phosphorylation events on the quantitative modulation of signal propagation, since a deletion of one of the two functionally redundant genes has no phenotype. One proven genetic approach is to delete the second functionally redundant gene (here it would be *DIG2*), and examine the effect of mutations in the first protein in the deleted strain background. However, this approach is not suitable for all experimental systems and may result in pleiotropic effects due to a lack of complete redundancy. Here, we chose to investigate the suitability of using a sensitized genetic background (a background that reveals a phenotype that would not be clear in a wild type background). For this purpose we turned to a strain that was constructed to measure the Dig1-Ste12 interaction *in vivo* in single cells using FRET (fluorescence resonance energy transfer) [13]. The strain contained YFP-tagged Dig1 that can be co-expressed with CFP-tagged Ste12, and we additionally engineered this strain to contain the P*_PRM1_*-mCherry reporter to simultaneously measure pheromone pathway output.

Since the peptide V266-K282 contained four serine and two threonine residues, we chose to introduce two separate 3X Dig1-YFP mutant constructs (S269A, S272A, S275A and T277A, S279A, T280A) as well as individual Dig1-YFP point mutants into the *DIG1* chromosomal locus under the control of the native *DIG1* promoter. We verified the comparable expression level of the mutant proteins in each resulting strain by immunoblot and their nuclear localization by fluorescence microscopy (Figure 4B and Figure S6) [36].

The reference strain containing CFP-Ste12 and Dig1-YFP showed robust pheromone-dependent transcriptional induction as measured by quantitative fluorescence microscopy (Figure 4C). Surprisingly, however, CFP-Ste12 showed a severely impaired transcriptional response in a *dig1*? background (Figure 4C). In the absence of the CFP tag, untagged Ste12 is sufficient to activate pheromone target genes in a *dig1*? background [44]. However, when tagged on its N-terminus, CFP-Ste12 required Dig1 as a co-activator. While normally Dig1 acts as a repressor to Ste12, by N-terminally tagging Ste12 with a fluorescent protein, we effectively reversed the regulatory relationship of Ste12 with its binding partner Dig1. This novel reliance on Dig1 for pheromone-induced transcriptional activation is consistent with recent work showing that Dig1 and Dig2 play positive roles in the pheromone response [45]. The sensitized CFP-Ste12, Dig1-YFP background allowed us to monitor the effect of mutations of putative phosphorylation sites on Dig1 on pathway output, in the presence of Dig2.

### Closely situated Dig1 mutations have opposing effects on pathway output

We took advantage of this increased reliance on Dig1 for pheromone output to explore novel signaling phenotypes of phosphorylation site mutants. We first measured P*_PRM1_*-mCherry fluorescence in cells treated with a high dose of 20 nM α-factor. Output from the first 3X mutant strain (Dig1-YFP^S269A, S272A, S275A^) was indistinguishable from reference cells (Figure S7). By contrast, output from the second 3X mutant (Dig1-YFP^T277A, S279A, T280A^) was severely diminished, similar to the *dig1*? strain (Figure 4C).

Strains that carried individual mutations of T277, S279, or T280 each showed altered pathway output compared to wild type Dig1-YFP. Output from Dig1^T280A^ was diminished compared to the reference strain, similar to the 3X mutant strain (Figure 4C). However, in the T277A and S279A strains, output was significantly higher than the reference strain (Figure 4C). These phenotypes persisted across a dose response (Figure 4D). Surprisingly site-specific mutation of closely situated residues within Dig1-YFP had opposite effects on system output.

### Modeling complex Dig1 phospho-regulation constrains possible pathway architectures

To develop a better understanding of the opposing roles of the Dig1 mutations in the context of the novel co-activator role of Dig1-YFP, we again turned to computational modeling. The Dig1 model treated Dig1 as an activator of Ste12, and incorporated the constraint that the T280A mutation was dominant over the T277A and S279A mutations since the T280A phenotype masked the T277A and S279A phenotypes in the 3X mutant. Dig1 residues T277, S279 and T280 are not likely to be MAPK substrates. Thus, developing a model for Dig1 required exploration of several possible pathway architectures.

We constructed and studied four different models: 1) in which all phosphorylation was induced by pheromone; 2) in which all sites were initially phosphorylated and dephosphorylation was pheromone-dependent; 3) in which T280 was phosphorylated in response to pheromone while T277/S279 were dephosphorylated in response to pheromone; and 4) in which all phosphorylation was constitutive and independent of pheromone (Figure 4E, 4G, and Figure S8). We studied the behavior of the four models as a function of pheromone concentration. We were unable to recapitulate the opposing single-mutant phenotypes with either a constitutive phosphorylation model (model 2) or a model in which all sites were dephosphorylated after pheromone induction (model 4) (Figure S8). However, both the model in which all the sites were phosphorylated in response to pheromone (model 1), and the model in which T280 was phosphorylated in response to pheromone while T277/S279 were dephosphorylated in response to pheromone (model 3), satisfied the experimental constraints (Figure 4F and 4H). While model 1 fit the data over a larger range of parameters, model 3 better reproduced the hypersensitivity of T277A at low doses of pheromone (Figure 4F, 4H, Figure S9, and Figure S10). Thus, for these models to recapitulate the experimental results, T280 must be phosphorylated in response to pheromone, while at the same time T277/S279 must either be phosphorylated or dephosphorylated in response to pheromone.

## Discussion

While many sites of phosphorylation have been mapped in proteomic studies from mammals, invertebrates and yeast, the vast majority of sites have no known function. We hypothesized that many of these sites are likely to exert dynamic regulatory roles in signaling pathways, the effects of which can only be revealed with quantitative assays in the context of the specific stimulus about which they convey information. We therefore developed a systematic, general approach to prioritize the study of individual phosphorylation events and their potential functions in signaling networks, and demonstrated the utility of our approach on components of the pheromone response system in the budding yeast *Saccharomyces cerevisiae*.

Since the approach described here requires targeted mutagenesis of genomic DNA, which is straightforward to accomplish in genetically tractable model organisms like yeast, we acknowledge that this methodology is more challenging to implement in other organisms. However with recent advances in tools like zinc finger nucleases and TALENs it will become increasingly possible to target mutations in many diverse organisms and cell types [46]. We believe that the approach that we have employed here will soon be applicable to, e.g., iPS cells.

We focused on three non-kinase proteins with distinct signaling roles upstream and downstream of the protein kinase cascade (Figure 1): 1) Ste12, a transcription factor activated by the MAP kinase cascade that induces genes involved in mating, 2) Ste50, an adaptor protein that acts upstream of the MAP kinase cascade to link the G protein-associated rho-like GTPase (Cdc42)-PAK kinase (Ste20) complex to the MAP3K (Ste11) and, 3) Dig1, a protein involved in regulating the activity of Ste12. Using quantitative single cell assays we showed that 6 phosphorylation sites on 4 separate motifs in 3 signaling components (Ste12, Ste50 & Dig1) quantitatively affected pheromone pathway output. While the most parsimonious interpretation of the quantitative phenotypes we observed is that the changes in pathway output are the direct consequence of the inability of the mutated residue to be phosphorylated, we have not ruled out that the altered pathway output we observed is due to structural changes unrelated to phosphorylation and/or altered protein/protein interactions caused by the alanine substitutions.

In strains expressing a protein with phosphorylation site point mutations, no cell will have the mutated site phosphorylated. In the reference strain in any given single cell, the fraction of the population of molecules of the protein with the site phosphorylated is unknown. We can thus expect that the effect of any phosphorylation site on signaling pathway output will be incompletely penetrant. The quantitative phenotypes we measured in the mutant strains – measurements made comparing the population of mutant cells to the heterogenous reference cells – may therefore underestimate the effect that these phosphorylation sites exert on the activity of the proteins they modify.

On Ste12, we identified 2 putative phosphorylation sites, S400 and T525, that each contribute ∼25% to the transcriptional activity of Ste12 across a dose response of pheromone (Figure 2D). Based on neighboring sequence context, these sites are likely to be MAPK targets and we demonstrated that they both contribute to the appropriate transcriptional output to a given dose of pheromone. The conservation of these sites among closely related yeast species and the quantitative agreement of the computational model with the experimental results lend credence to this notion (Figure 2). While the 25% gain in activity afforded by these sites is unlikely to be absolutely required for mating in laboratory settings, such a quantitative increase in output may have conferred competitive fitness over evolutionary timescales.

The adaptor protein Ste50 links the MAP3K Ste11 to active Cdc42 and Ste20, thereby localizing Ste11 to its upstream activators at the plasma membrane. Ste11 then signals through different MAPK cascades via its association with different scaffold proteins: Ste5 directs signaling to the pheromone pathway, while Pbs2 directs signaling to the hyper-osmotic stress pathway. Several groups have suggested that phosphorylation of Ste50, possibly on S202, may help determine how much signal from active Ste11 goes to the pheromone pathway, and how much goes to the hyper-osmotic stress pathway [47], [48], [49]. Mechanistically, phosphorylation of Ste50 on S202 may influence pathway choice by regulating protein-protein interactions with membrane anchoring factors that associate with the pathway-specific scaffolds [47], [49]. Here we propose that phosphorylation of S202 on Ste50 anchors a negative feedback loop that inhibits pheromone pathway output in response to low doses of pheromone (Figure 3). Thus, phosphorylation of S202 may serve to dampen the threshold required to activate a full-fledged pheromone response. This negative feedback loop could be relevant when mating partners are scarce and commitment to the mating program would waste resources, and also when both pheromone and high osmolarity are present to ensure that enough signaling bandwidth is available to trigger the response to osmotic stress.

For the redundant repressor protein Dig1, we utilized a sensitized genetic background in which CFP-tagged Ste12 requires Dig1 for its full activity to increase the likelihood of revealing functional roles for Dig1 mutants without having to delete the paralogous repressor Dig2. We identified 3 putative sites on Dig1 that alter the pheromone response. T280 is required for Dig1-mediated Ste12 activity: cells expressing Dig1-YFP^T280A^ displayed a severely diminished transcriptional response, phenocopying the full deletion of Dig1. Strikingly, the closely situated residues T277 and S279 had the opposite effect in that they inhibit signal output: mutation of either residue leads to an increased pheromone response. Since these sites lack consensus MAPK sequence context, the mechanistic and architectural details of how they might exert their effects could be constrained through computational modeling, but not determined unequivocally. Similarly, since in this sensitized strain background, CFP-Ste12 required Dig1-YFP for full activity, the significance of the phenotypes of the Dig1 mutations for the native pheromone pathway remains to be determined. However, our current findings that closely situated mutations exert opposite effects are sufficient to suggest that multiple layers of post-translational regulation can be superimposed to fine-tune quantitative signal output.

Our results indicate that many putative sites of phosphorylation contribute to and adjust the input-output relationship of this model eukaryotic signaling system. We propose that multiple small influences of such individual phosphorylation events can endow signaling systems with plasticity and evolvability. Consistent with this view, in the motifs that we studied on Ste12 (S400, T525) and Ste50 (S202), the presence of a minimum consensus requirement for a MAPK substrate (serine or threonine preceded by a proline residue) arose relatively recently and is conserved in closely related yeast species but not in more distantly related yeast species (Figures 2A and 3A). In Dig1, where residues T277, S279 and T280 are not likely to be MAPK substrates, the presence of serine or threonine residues at similar positions in orthologs is conserved in closely related yeast species but not in more distantly related yeast species (Figure 4A). In all cases, the putative phosphorylation events that we studied fall in regions predicted to be unstructured in Ste12, Ste50 and Dig1. Such unstructured regions are well suited to accommodate amino acid changes to generate new sites for post-translational events like phosphorylation [50], [51], [52], [53], [54]. This plasticity and evolvability may be advantageous for fine-tuning the input/output relationship of the pathway. By contrast, the core activation mechanism of the pathway is highly conserved during evolution. Activation of the MAPK Fus3 is governed by a dual phosphorylation event (T180, Y182) and mutation of either site results in a complete loss of function [9]. This dual phosphorylation motif in the kinase activation loop is conserved in MAPKs all the way from yeast to mammalian MAPKs such as Erk1 and Erk2 [9].

In conclusion, we suggest that new layers of post-translational regulation can be gained and lost to rapidly adapt quantitative system output in the face of changing selective pressure without compromising the core structural and functional integrity of key signaling proteins. We note that this idea suggests means to systematically design and alter signaling pathway components to introduce novel regulatory loops or sever existing ones, and to confer new regulatory properties to pathway specific kinases and phosphatases. An approach based on iterating the design and construction of such re-engineered signaling pathways guided by quantitative experimentation and interpreted via appropriate models, should facilitate design-based alteration of signaling systems to bring about desired cellular behaviors.

## Materials and Methods

### Cell growth and reagents

Routine growth of yeast strains was performed as described (*1*). Strains were grown on YAPD plates (YPD with supplemental adenine) and/or YAPD liquid (YPD with supplemental adenine) media. Yeast deletions were selected on YAPD/HygB or YAPD/Kan plates. Mutant strains were grown on SD plates and/or SD liquid media (yeast nitrogen base, 2% glucose, with appropriate selection for auxotrophic markers).

### Pheromone synthesis

Alpha factor (or pheromone; Trp-His-Trp-Leu-Gln-Leu-Lys-Pro-Gly-Gln-Pro-Met-Tyr) was ordered from and synthesized at the W.M. Keck Foundation Biotechnology Resource Laboratory (Yale University). We made a 1 mM stock solution and stored it in aliquots at -80°C.

### Pheromone treatment

For large-scale cell growth cells were treated with 1 µM pheromone for the time indicated. For both microscopic and flow cytometric studies cells were treated with 1-20 nM pheromone as indicated for the described time.

#### Pheromone sensitivity assays

We qualitatively tested the pheromone sensitivity of all reference strains, deletion strains and phosphorylation site mutants. We prepared YAPD plates with 10-fold dilutions of pheromone, ranging from 1 µM to 1 nM. We diluted saturated overnight cultures of strains to OD_600_ = 1.0. On each of the four plates we spotted 10, 100 and 1000 cells of each strain and incubated the plates 30°C for 20 hrs, following which the growth of cells was recorded.

### Antibodies

We used custom rabbit polyclonal antibodies against pheromone pathway proteins (*2*).We monitored phosphorylation of the MAPKs (Fus3 and Kss1) in populations of cells by Western blot analysis with rabbit anti-phospho-p44/42 antibodies (Cell Signaling Technologies, Beverly, MA). We monitored the appearance of fluorescent protein fusion proteins by Western blot analysis using mouse monoclonal anti-GFP antibodies (JL-8) (BD Biosciences, Palo Alto, CA). We verified the relative abundance of cell extract per gel lane by Western blot analysis using a mouse monoclonal antibody against the reference protein, GAPDH (Abcam, Cambridge MA).We used one of two fluorescently-labeled secondary antibodies to visualize all results on Westerns (Alexa Fluor^®^ 680 series, Invitrogen Co., Carlsbad, CA; IRDye^®^ 800 series, Rockland Immunochemicals Inc., Gilbertsville, PA).

### Construction of deletion strains

We created yeast strains lacking the ORFs *STE12*, *DIG1* and *STE50*. STE12: We deleted *STE12* from ACLY379 using PCR-mediated one-step replacement (*1*) with the pFA6a-kanMX6 template (*3*) and primer pair STE12KOf/STE12KOr, creating D12. We verified the deletion by PCR and Western blot analysis. Subsequently, we replaced the *PRM1* ORF in D12 with a PCR product containing the *mCHERRY* coding sequence, the *ADH1* terminator and the *hph* gene from pAG32-hphMX6 (*4*), creating DPY112. DIG1: We deleted *DIG1* by one-step excision of *ura3*-marked *DIG1-YFP* from RCY1130 using 5-FOA (*1*) to select for excision products, creating RCY2005. We verified the deletion using epifluorescent microscopy and Western blot analysis. Subsequently, we replaced the *PRM1* ORF in RCY2005 with a PCR product containing the *mCHERRY* coding sequence, the *ADH1* terminator and the *hph* gene from pAG32-hphMX6 (*4*) creating RCY2005pch. STE50: We deleted *STE50* from TCY3154 using PCR-mediated one-step replacement (*1*) with the pAG32-hphMX6 (*4*) template and primer pair STE50KOf/STE50KOr, creating DPY250. We verified the deletion by PCR and Western blot analysis.

### Construction of strains containing mutant proteins

We created yeast strains with mutant alleles of *STE12*, *DIG1-YFP* and *STE50*. To make the mutant *STE12* strains, we amplified the wild type STE12 gene (including 968 bp of endogenous promoter, the ORF and 634 bp of endogenous terminator) from W303a genomic DNA and cloned this PCR product into pRS406 at XhoI and EcoRI sites, creating pSTE12-406. We performed site-directed mutagenesis with the GeneTailor™ site directed mutagenesis kit (Invitrogen Co., Carlsbad, CA) using the following primer pairs: STE12M3f/STE12M3r, STE12-s400a-f/STE12-s400a-r, STE12-s402a-f/STE12-s402a-r, STE12-t405a-f/STE12-t405a-r, STE12-s406a-f/STE12-s406a-r and STE12M4f/STE12M4r and methylated pSTE12-406 as the template, creating pSTE12m3-406, pSTE12s400a-406, pSTE12s402a-406, pSTE12t405a-406, pSTE12s406a-406, and pSTE12m4-406. The sequences of the mutated plasmids were verified (MWG Biotech, High Point, NC). We linearized the mutant plasmids in the STE12 promoter region with BsiWI (New England Biolabs, Beverly, MA), and individually transformed them into DPY112 and plated on SD plates lacking uracil, creating DPY1203, CRY1004, CRY1005, CRY1006, CRY1007 and DPY1204. We verified the presence of and normal abundance of Ste12^S400A,S402A,T405A,S406A^, Ste12^S400A^, Ste12^S402A^, Ste12^T405A^, Ste12^S406A^, and Ste12^T525A^ proteins using Western blot analysis and the absence of the Ste12 protein in the Ste12D reference strain.

To make the *DIG1-YFP* mutant strains, we performed site-directed mutagenesis and sent for sequencing as above, using primer pairs DIG1M1f/DIG1M1r, DIG1M3f/DIG1M3r, Dig1-t277a-f/Dig1-t277a-r, Dig1-s279a-f/Dig1-s279a-r, and Dig1-t280a-f/Dig1-t280a-r and methylated pDIG1YFP-406 (*3*) template, creating pDIG1m1YFP-406, pDIG1m3YFP-406, pDIG1t277aYFP-406, pDIG1s279aYFP-406, and pDIG1t280aYFP-406. We linearized the mutant plasmids in the *DIG1* promoter region with BstEII (New England Biolabs, Beverly, MA), individually transformed them into RCY2005pch and plated on SD plates lacking uracil, creating DPY1001, DPY1003, TCY3328, TCY3329, and TCY3330. We verified both the presence and normal abundance of Dig1^S126A,S127A,S129A^, Dig1^T277A,S279A,T280A^-YFP, Dig1^T277A^-YFP, Dig1^S279A^-YFP, and Dig1^T280A^-YFP proteins by Western blot analysis and the absence of the Dig1 protein in the Dig1D reference strain. We additionally verified that the Dig1-YFP proteins localized to the nucleus using epifluorescent microscopy.

To make the *STE50* mutant strains, we amplified the wild type STE50 gene (including 76 bp of endogenous promoter, the ORF and 384 bp of endogenous terminator) from W303 genomic DNA and cloned the PCR product into pRS406 at the XhoI site, creating pSTE50-406. We performed site-directed mutagenesis and sent for sequencing as above, using primer pairs STE50M1f/STE50M1r, STE50-s202a-f/STE50-s202a-r, and STE50-t205a-f/STE50-t205a-r and methylated pSTE50-406 as the template, creating pSTE50m1-406, pSTE50s202a-406, and pSTE50t205a-406. We linearized the mutant plasmids in the STE50 promoter region with PshAI (New England Biolabs, Beverly, MA), transformed into DPY250 and plated on SD plates lacking tryptophan and uracil, creating DPY5001, TCY3344.2, and TCY3345.2. We verified the presence and normal abundance of Ste50^S202A,T205A^, Ste50^S202A^, and Ste50^T205A^ proteins by Western blot analysis and the absence of the Ste50 protein in the Ste50D reference strain.

### Fluorescent transcription assays of phosphorylation mutant strains

We quantitatively tested the pheromone response of all strains by measuring FP fluorescence in single cells as a surrogate for transcriptional output from a pheromone responsive promoter (P_PRM1_-YFP). Starting from single colonies on YAPD plates, we inoculated 4 mL cultures in YAPD. We monitored growth by A_600_ and diluted into 4 mL SDC at the end of the day to obtain mid-log phase cultures in the morning. We diluted cultures to OD_600_ = 0.2 in YAPD and grew to OD_600_ = 0.8. We centrifuged 1.5 mL cells for 30 sec at 15,000 rpm, resuspended in 1.5 mL SDC with 40 µg/mL casein (from DIG nucleic acid detection kit, Roche Diagnostics Corporation, Indianapolis, IN) and sonicated the cells to disperse clumps. We pipetted 500 µL of each strain into a 96 well deep well plate for the untreated (no pheromone) samples and added 50 µL 50 µg/mL cycloheximide (Calbiochem (EMD), San Diego, CA). We pipetted 495 µL of each strain into the same 96 well deep well plate and added 5 µL of 100 µM pheromone in SDC/casein. We incubated the plate at 30°C for 15 minutes with shaking (300 rpm) and stopped the experiment with 50 µL 50 µg/mL cycloheximide in SDC/casein (Calbiochem (EMD), San Diego, CA). We incubated an additional 2.5 hrs at 30°C, shaking at 300 rpm, to allow for fluorophore maturation. We sonicated the cells again, diluted 1:20 in SDC/casein/cycloheximide, pipetted 200 µL into 96 well glass bottom plates and let the cells settle for 10 minutes. We performed optical microscopic cytometry and image capture as described elsewhere (*5, 6*) and image analysis, and data processing using the open source software packages Cell-ID and PAW (*7, 8*).

We compared pathway output in ACLY379pch, DPY112, DPY1203, CRY1004, CRY1005, CRY1006, and CRY1007. Starting from single colonies, we inoculated 4 mL cultures in SDC (ACLY379pch and DPY112) or SD/-U (DPY1203, CRY1004, CRY1005, CRY1006, and CRY1007) and grew over the course of the day to obtain log phase cells. We diluted overnight 4 mL cultures in the same media to obtain mid-log phase cells in the morning, at which point we adjusted to OD_600_ = 0.25 and allowed the cells to grow for one generation (90 minutes). We prepared a 4-fold dilution series of pheromone in SDC/casein ranging from 20 nM - 0.31 nM. We aliquoted 7×0.5 mL of each dilution into a 96-well deep well plate, and to another set of 7 wells we added 0.5 mL SDC/casein/cycloheximide in order to measure baseline pathway output. We sonicated 1.0 mL of each culture to disperse cell clumps and added 50 µL of each culture to the wells containing SDC/casein/cycloheximide and the pheromone-dilution series. We incubated the plate at 30°C for 3 hours, shaking at 300 rpm, at which point we stopped the experiment and allowed the fluorophores to mature as described above. We sonicated the plate and quantified reporter fluorescence by microscopy (3.1.a) and flow cytometry. 5 ul of cells was subjected to flow cytometric analysis using a BD LSR-II flow cytometer (UCSF QB3 core facility) equipped with a high throughput sampler, a 488 nm 100 mW laser, FITC emission filter and FACS DIVA software to compile .fcs files.

We compared pathway output in RCY1130pch, RCY2005pch, DPY1003, TCY3328, TCY3329, and TCY3330. Starting from single colonies, we inoculated 4 mL cultures in SDC (RCY1130pch), SD/-W (RCY2005pch), or SD/-U (DPY1003, TCY3328, TCY3329, and TCY3330) and grew over the course of the day to obtain log phase cells. We diluted overnight 4 mL cultures in the same media to obtain mid-log phase cells in the morning, at which point we adjusted to OD_600_ = 0.25 and allowed the cells to grow for one generation (90 minutes). We prepared a 4-fold dilution series of pheromone in SDC/casein ranging from 20 nM-0.31 nM. We aliquoted 6×0.5 mL of each dilution into a 96-well deep well plate, and to another set of 6 wells we added 0.5 mL SDC/casein/cycloheximide in order to measure baseline pathway output. We sonicated 1.0 mL of each culture to disperse cell clumps and added 50 µL of each culture to the wells containing SDC/casein/cycloheximide and the pheromone-dilution series. We incubated the plate at 30°C for 3 hours, shaking at 300 rpm, at which point we stopped the experiment and allowed the fluorophores to mature as described above. We sonicated the plate and quantified reporter fluorescence by microscopy (3.1.a).

We compared pathway output in TCY3154, DPY250, DPY5001, TCY3344.2, and TCY3345.2. Starting from single colonies, we inoculated 4 mL cultures in SD/-W (TYC3154 and DPY250), SD/-W-U (DPY5001, TCY3344.2, and TCY3345.2) and grew over the course of the day to obtain log phase cells. We diluted overnight 4 mL cultures in the same media to obtain mid-log phase cells in the morning, at which point we adjusted to OD_600_ = 0.25 and allowed the cells to grow for one generation (90 minutes). We prepared a 4-fold dilution series of pheromone in SDC/casein ranging from 20 nM-0.31 nM. We aliquoted 5×0.5 mL of each dilution into a 96-well deep well plate, and to another set of 5 wells we added 0.5 mL SDC/casein/cycloheximide in order to measure baseline pathway output. We sonicated 1.0 mL of each culture to disperse cell clumps and added 50 µL of each culture to the wells containing SDC/casein/cycloheximide and the pheromone-dilution series. We incubated the plate at 30°C for 3 hours, shaking at 300 rpm, at which point we stopped the experiment and allowed the fluorophores to mature as described above. We sonicated the plate and quantified reporter fluorescence by microscopy (3.1.a).

### Bioinformatic analysis

Previously determined sites of phosphorylation, protein domains, protein/protein interaction information and gene and protein sequences were obtained from primary literature, the Biobase YPD database [www.proteome.com] and the *Saccharomyces* genome database (S288C strain background) [www.yeastgenome.org].

We used tree-assisted ortholog alignments to identify *S. cerevisiae* pheromone pathway protein orthologs in other yeast species. In two cases (Fus3 and Kss1) orthologs from some species were not included in the ortholog assignments. In these cases we obtained the absent ortholog sequences from the *Y. lipolytica* database (*9*) from orthlogues assigned in (*10*) and from the Wapinski update (January 2009) that is available on SGD (yeastgenome.org) in the fungal orthogroups repository. We used the Yeast Gene Order Browser (YGOB) to distinguish orthologs and paralogs in cases of retained duplicates (*11*).

We aligned regions of *S. cerevisiae* Ste12, Dig1, and Ste50 to the best matching sequences in the orthologs identified by Wapinski et al. to demonstrate the conservation of motifs and phosphorylation sites.

### Computational modeling

For each protein of interest (Ste12, Dig1, Ste50), we wrote a set of ordinary differential equations (ODEs) to describe roles for phosphorylation sites in the context of pheromone signaling. All reaction equations are based on mass action kinetics, and a Hill function describes the transcriptional induction step. Although the models employ the standard mathematical approach of ODE-based mass action equations, the models qualitatively differ from standard approaches in two important ways.

First, instead of explicitly keeping track of concentrations of the species in physical units, the models represent the species in abstract, non-physical units of “activity”. These abstract species still exert their effects in proportion to their amounts, and thus still operate in a mass action paradigm. This method emphasizes the generic architectural constraints of the pathway and avoids the requirement of fitting specific unmeasured parameters.

Second, the models incorporate phosphorylation in a non-canonical way. Standard ODE models represent unmodified and modified proteins as distinct species that have their own production, decay and reaction rates. Here, in contrast, phosphorylation does not generate novel species with new properties, rather phosphorylation tunes the inherent activity of the native proteins by acting “in trans”. In this way, the models remain uncluttered by additional unmeasured parameters and assumptions, and therefore emphasize how the phosphorylation events tune the activity of the protein they modify. This approach could be used to study the role of any protein modification.

To implement the models, equations were coded and simulated across a pheromone dose response in MatLab R2008a using the ODE23s solver (code included as supplementary material). Parameters were all set to 1 to initiate the system, and were modified to fit the experimental data. Once a set of parameters was obtained that fit the experimental data, we performed sensitivity analysis by sweeping through 2- and 10-fold increases and decreases of each parameter and simulating the ratio of mutant to reference strain transcription in response to 20 nM pheromone (supplementary figures). The observed differences between the mutants and reference strain transcriptional responses were insensitive to most parameter changes. Sensitive parameters are discussed in the main text.

## Supporting Information

Figure S1 **Ste12^S402A^ and Ste12^T406A^ have the same dose response as wild type.** Cells bearing wild type or mutant Ste12 were treated with the indicated doses of pheromone and PRM1 driven mCherry was measured by flow cytometry.
(PDF)

Figure S2 **Modeling phosphorylation sites in trans.** The modeling strategy employed here treats the phosphorylation sites as separate entities from the proteins they modify (left). These phosphorylation sites (p sites) activate or inhibit the protein they modify (substrate) as a function of their concentration. In the abstracted example shown, reminiscent of the S202-mediated negative feedback loop, the effect of the phosphorylation site is the same whether “in trans” or as a “new species” – that is, a net negative effect on the activity of the MAPK. Advantages of the “in trans” model are that there is one fewer species and 4 fewer reaction rates to model. Moreover, there are fewer mechanistic assumptions in the “in trans” model, since the net inhibitory effect is all that is modeled. In the “new species” model, a mechanism must be specified for *how* the inhibitory effect happens (here it is shown as increasing the rate of MAPK dephosphorylation). The “in trans” formulation allows the models to be simplified, generic and easily modifiable without making mechanistic assumptions.
(PDF)

Figure S3 **Results of Ste12 modeling are robust to changes in parameters.** The strength of each parameter depicted in the cartoon model as well as the degradation constant for Ste12 were increased and decreased by 2-fold (top panels) and 10-fold (bottom panels), and the ratio of simulated output of wild type to S400A at 20 nM pheromone (the mutant phenotype) is plotted. The phenotype persists in the presence of 2-fold changes, but changes in magnitude with 10-fold changes to the parameters.
(PDF)

Figure S4 **Dose responses of Ste50^S202A,T205A^ and Ste50^T205A^.** Cells bearing mutant versions of Ste50 were treated with pheromone in the presence of 1-NM-PP1, imaged by epifluorescent microscopy and quantified using Cell-ID.
(PDF)

Figure S5 **Results of Ste50 modeling are robust to changes in parameters.** The strength of each parameter depicted in the cartoon model were increased and decreased by 2-fold (top panels) and 10-fold (bottom panels), and the ratio of simulated output of wild type to S202A at 20 nM pheromone (the mutant phenotype) is plotted. The phenotype persists in the presence of 2-fold and 10-fold changes.
(PDF)

Figure S6 **Dig1^T277A,S279A,T280A^ resides in the nucleus and does not destabilize Ste12.** Cells bearing CFP-Ste12 and either wild type or mutant Dig1-YFP were imaged in by epifluorescence microscopy. Single channel and merged images are shown.
(PDF)

Figure S7 **Dig1^S269A,S272A,S275A^ activates the pheromone response like wild type.** A. Alignment of the full tryptic peptide identified by mass spec as being phosphorylated against Dig1 orthologs in other yeast species. B. Cells bearing Dig1, Dig1^S269A,S272A,S275A^ , Dig1^T277A,S279A,T280^ and cells deleted for DIG1 treated with 20nM pheromone for 3hrs. Dig1^S269A,S272A,S275A^ is indistinguishable from wild type.
(PDF)

Figure S8 **Dig1 model 2 and model 4 do not fit the experimental data.** A. Cartoon depiction of Dig1 model 2, in which phosphorylation of T277 and T280 is constitutive and independent of pheromone. In this model, in order to have a dose dependent increase in Dig1 activity in the presence of constitutive phosphorylation of T277 and T280, we inserted a pheromone bypass directly on Dig1. B. Simulation of Dig1 model 2: To agree with the wild type response, the strength of this bypass was such that the contribution of phospho-T277 and -280 were negligible. Thus the mutants are identical to the wild type. C. Cartoon depiction of Dig1 model 4, in which dephosphorylation of both T277 and T280 is pheromone-dependent. D. Simulation of Dig1 model 4: The strength of the influence of each phosphorylation site had to be so strong, such that if one site was mutated, the effect of the other dominated over the pheromone response. Thus, the mutants cannot recapitulate the experimental dose responses.
(PDF)

Figure S9 **Dig1 model 1 is robust to 2-fold changes in parameters that determine the influence of the phosphorylation sites.** The strength of each parameter depicted in the cartoon model were increased and decreased by 2-fold (top panels) and 10-fold (bottom panels), and the ratios of simulated output of wild type to T277A (black) and wild type to T280A (red) at 20 nM pheromone are plotted. The T277A phenotype persists (within pink boxes) with 2-fold changes to every parameter. The T280A phenotype persists except when the strength of the the Dig1-independent pheromone bypass (B4) becomes too strong. 10-fold changes to any parameter significantly change the simulation results.
(PDF)

Figure S10 **Dig1 model 3 is robust to 2-fold changes in parameters that determine the influence of the phosphorylation sites.** The strength of each parameter depicted in the cartoon model were increased and decreased by 2-fold (top panels) and 10-fold (bottom panels), and the ratios of simulated output of wild type to T277A (black) and wild type to T280A (red) at 20 nM pheromone are plotted. The T277A phenotype persists (within pink boxes) with 2-fold changes to every parameter. The T280A phenotype persists except when the strength of the the Dig1-independent pheromone bypass (B5) becomes too strong. 10-fold changes to any parameter significantly change the simulation results.
(PDF)

Table S1 **Strains used in this study.**
(DOCX)

Table S2 **Primers used for strain construction and mutagenesis.**
(DOCX)

Table S3 **Plasmids used in this study.**
(DOCX)

Table S4 **Public database links to phosphopeptides analyzed in this study.**
(DOCX)

Table S5 **P-values for mutant phenotypes.**
(DOCX)

Materials and Methods S1 (DOCX)

Text S1 **Matlab code for simulations.**
(DOCX)
