# Correction: Assigning Quantitative Function to Post-Translational Modifications Reveals Multiple Sites of Phosphorylation That Tune Yeast Pheromone Signaling Output

**DOI:** 10.1371/annotation/06dfa4e4-30f5-4d37-8559-0f2a9d11f0de

**Published:** 2013-06-28

**Authors:** David Pincus, Christopher J. Ryan, Richard D. Smith, Roger Brent, Orna Resnekov

In the Materials and Methods section of the article, the references were not presented properly. They appear in parentheses instead of brackets in the body of the article, and the references themselves did not appear.

The list of these references are available here: http://www.plosone.org/corrections/pone.0056544.001.cn.tif

There were multiple places in the article where the term "dig1ã" should have been "dig1∆".

There were two locations in the MAterials and MEthods section where the terms "Ste12D" and "Ste50D" should have been "ste12∆" and "ste50∆", respectively. 

