# Supplementary figures and images for: Assigning Quantitative Function to Post-Translational Modifications Reveals Multiple Sites of Phosphorylation That Tune Yeast Pheromone Signaling Output
Source: PLoS One. 2013 Mar 12;8(3):e56544. doi: 10.1371/journal.pone.0056544 (PMC3595240; doi:10.1371/journal.pone.0056544)

Figure S1

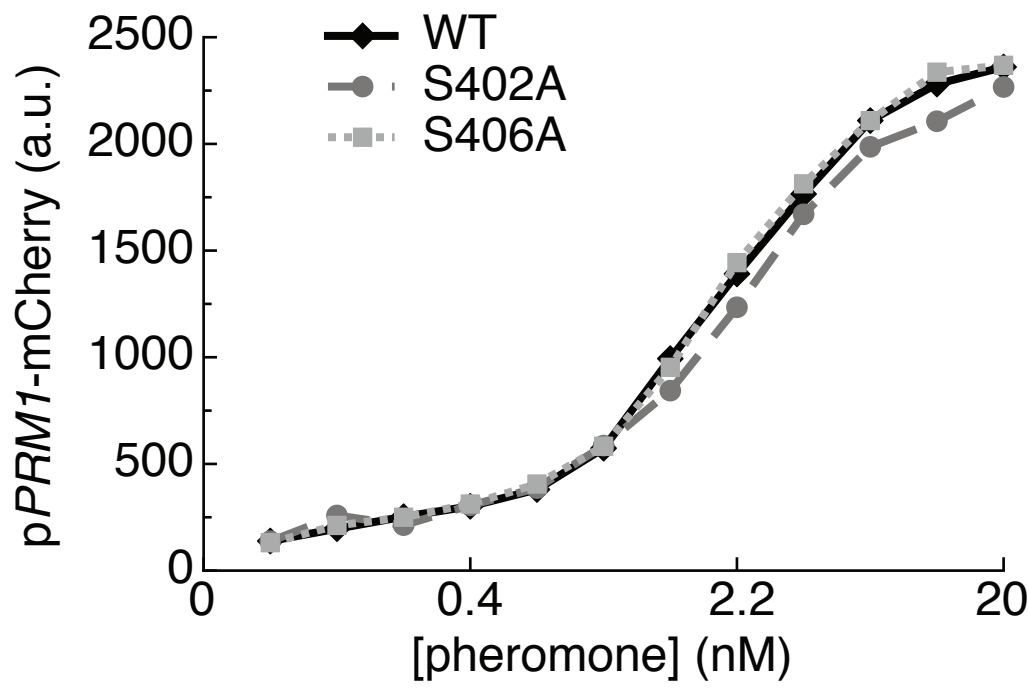

Supplement: Figure S1 — Ste12S402A and Ste12T406A have the same dose response as wild type. Cells bearing wild type or mutant Ste12 were treated with the indicated doses of pheromone and PRM1 driven mCherry was measured by flow cytometry. (PDF) [file pone.0056544.s001.pdf]

Figure S2

“in trans” model  
of phosphorylation

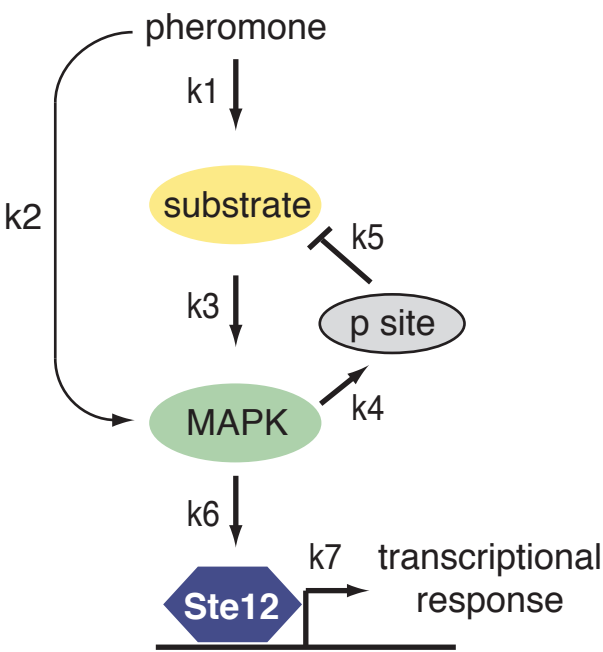

“new species” model  
of phosphorylation

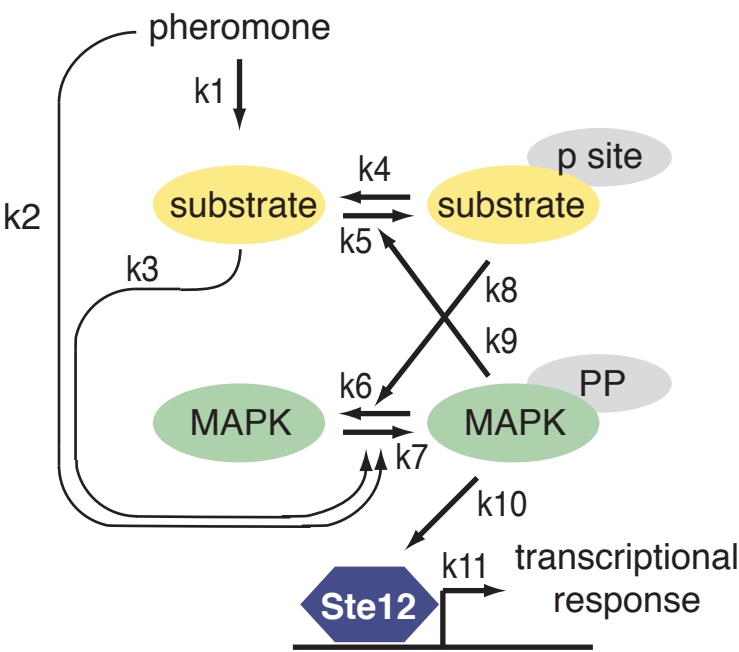

Supplement: Figure S2 — Modeling phosphorylation sites in trans. The modeling strategy employed here treats the phosphorylation sites as separate entities from the proteins they modify (left). These phosphorylation sites (p sites) activate or inhibit the protein they modify (substrate) as a function of their concentration. In the abstracted example shown, reminiscent of the S202-mediated negative feedback loop, the effect of the phosphorylation site is the same whether “in trans” or as a “new species” – that is, a net negative effect on the activity of the MAPK. Advantages of the “in trans” model are that there is one fewer species and 4 fewer reaction rates to model. Moreover, there are fewer mechanistic assumptions in the “in trans” model, since the net inhibitory effect is all that is modeled. In the “new species” model, a mechanism must be specified for how the inhibitory effect happens (here it is shown as increasing the rate of MAPK dephosphorylation). The “in trans” formulation allows the models to be simplified, generic and easily modifiable without making mechanistic assumptions. (PDF) [file pone.0056544.s002.pdf]

Figure S3

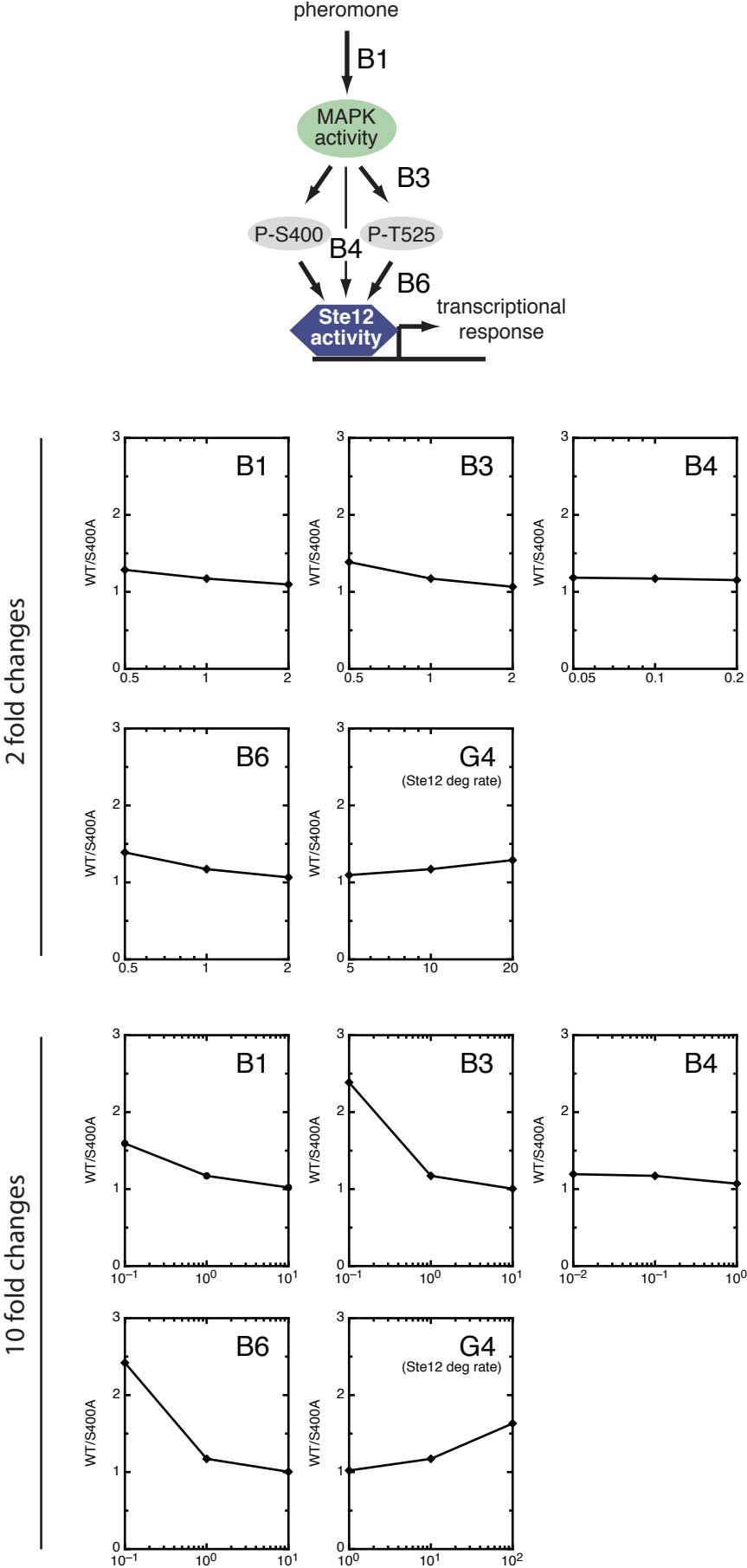

Supplement: Figure S3 — Results of Ste12 modeling are robust to changes in parameters. The strength of each parameter depicted in the cartoon model as well as the degradation constant for Ste12 were increased and decreased by 2-fold (top panels) and 10-fold (bottom panels), and the ratio of simulated output of wild type to S400A at 20 nM pheromone (the mutant phenotype) is plotted. The phenotype persists in the presence of 2-fold changes, but changes in magnitude with 10-fold changes to the parameters. (PDF) [file pone.0056544.s003.pdf]

Figure S4

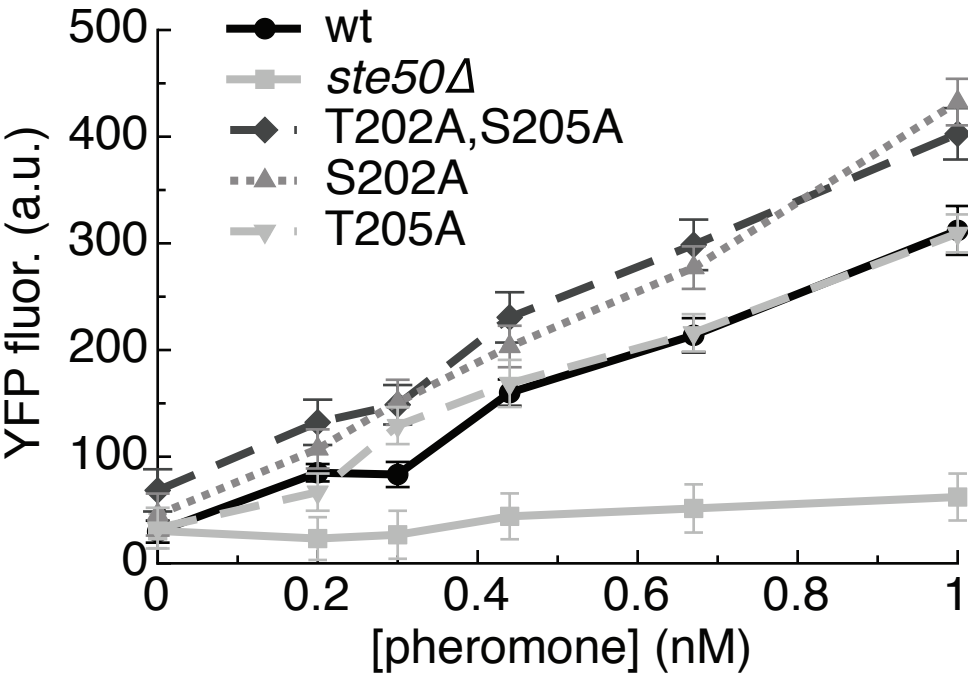

Supplement: Figure S4 — Dose responses of Ste50S202A,T205A and Ste50T205A. Cells bearing mutant versions of Ste50 were treated with pheromone in the presence of 1-NM-PP1, imaged by epifluorescent microscopy and quantified using Cell-ID. (PDF) [file pone.0056544.s004.pdf]

Figure S5

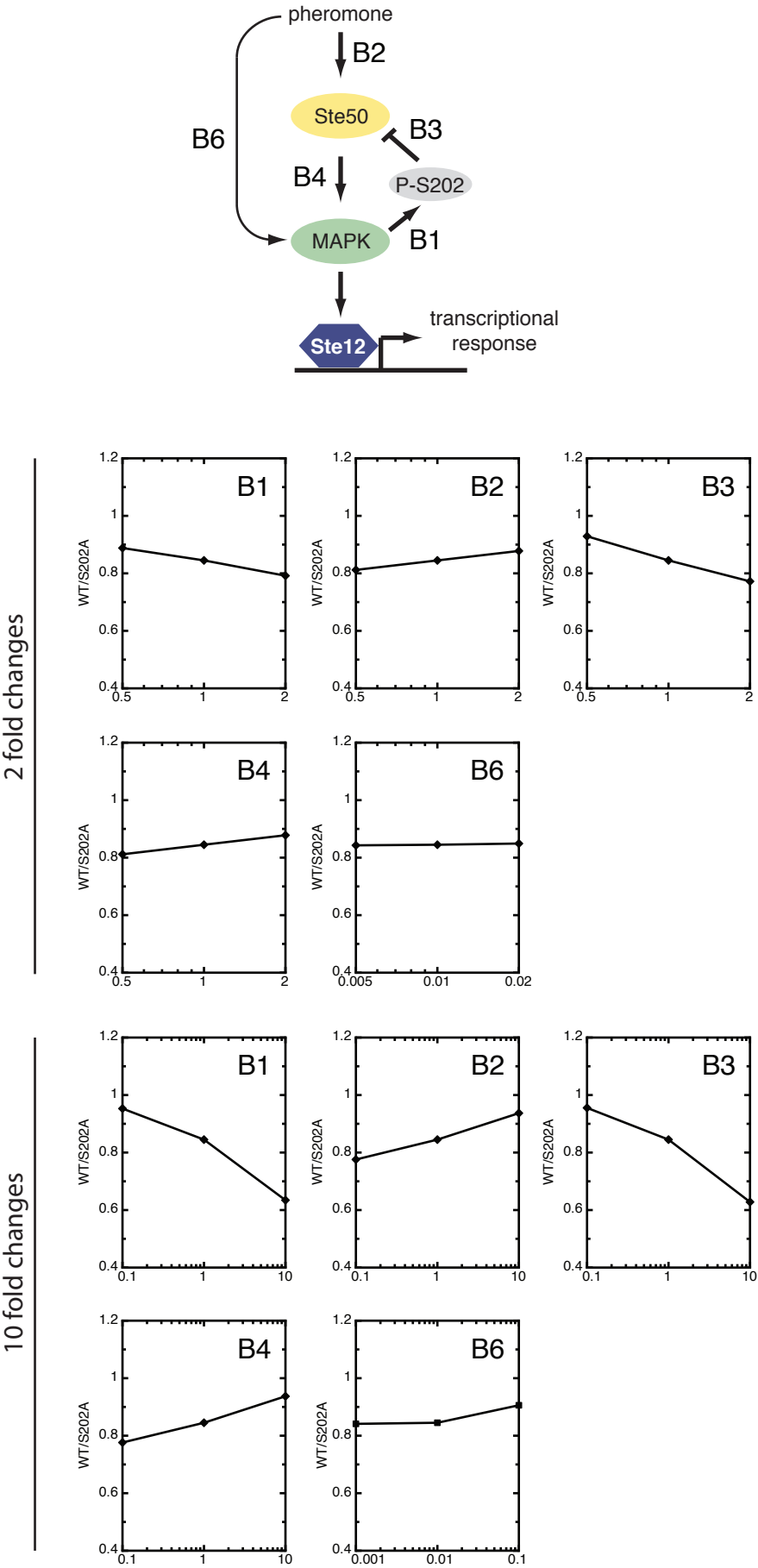

Supplement: Figure S5 — Results of Ste50 modeling are robust to changes in parameters. The strength of each parameter depicted in the cartoon model were increased and decreased by 2-fold (top panels) and 10-fold (bottom panels), and the ratio of simulated output of wild type to S202A at 20 nM pheromone (the mutant phenotype) is plotted. The phenotype persists in the presence of 2-fold and 10-fold changes. (PDF) [file pone.0056544.s005.pdf]

Figure S6

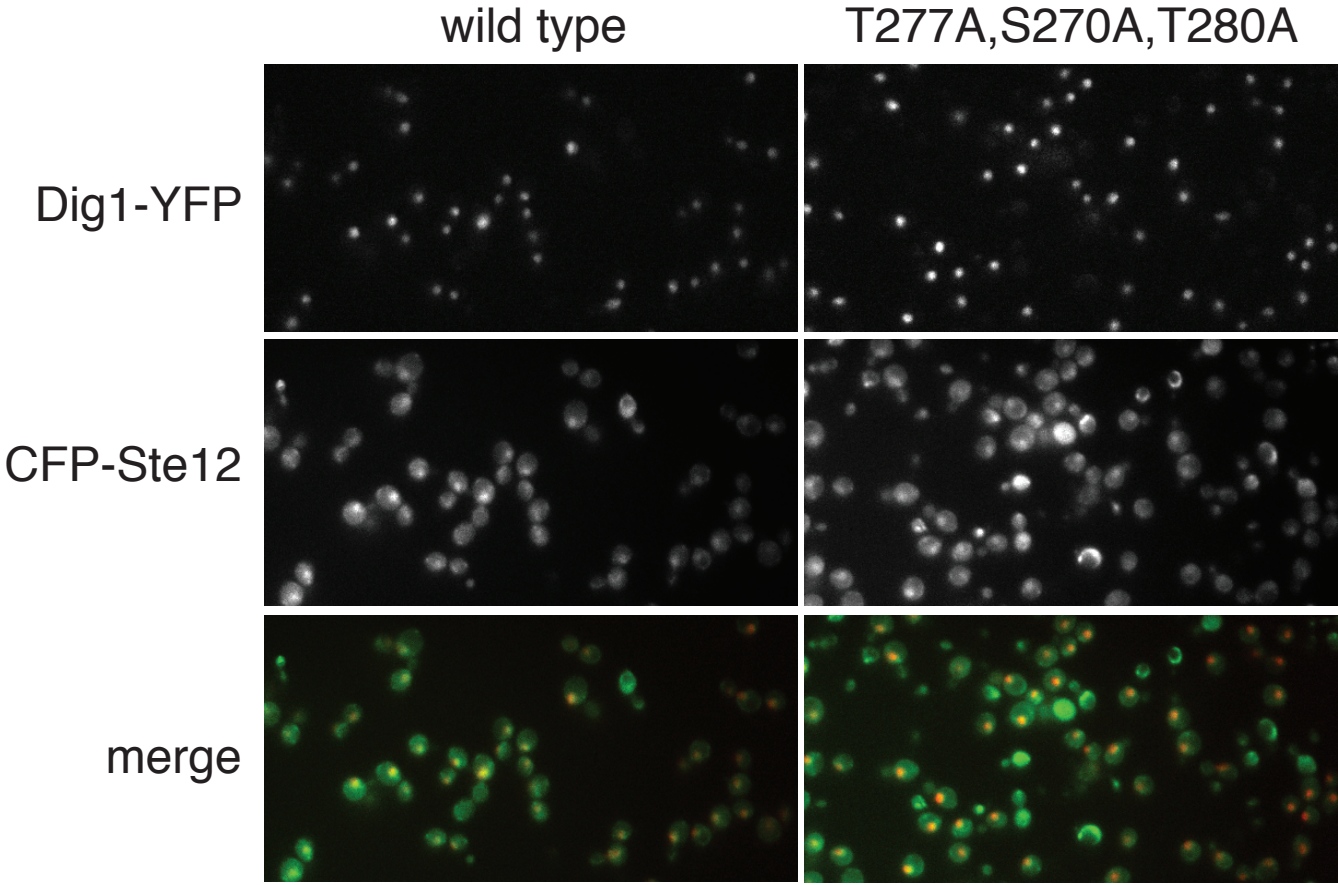

Supplement: Figure S6 — Dig1T277A,S279A,T280A resides in the nucleus and does not destabilize Ste12. Cells bearing CFP-Ste12 and either wild type or mutant Dig1-YFP were imaged in by epifluorescence microscopy. Single channel and merged images are shown. (PDF) [file pone.0056544.s006.pdf]

Figure S8

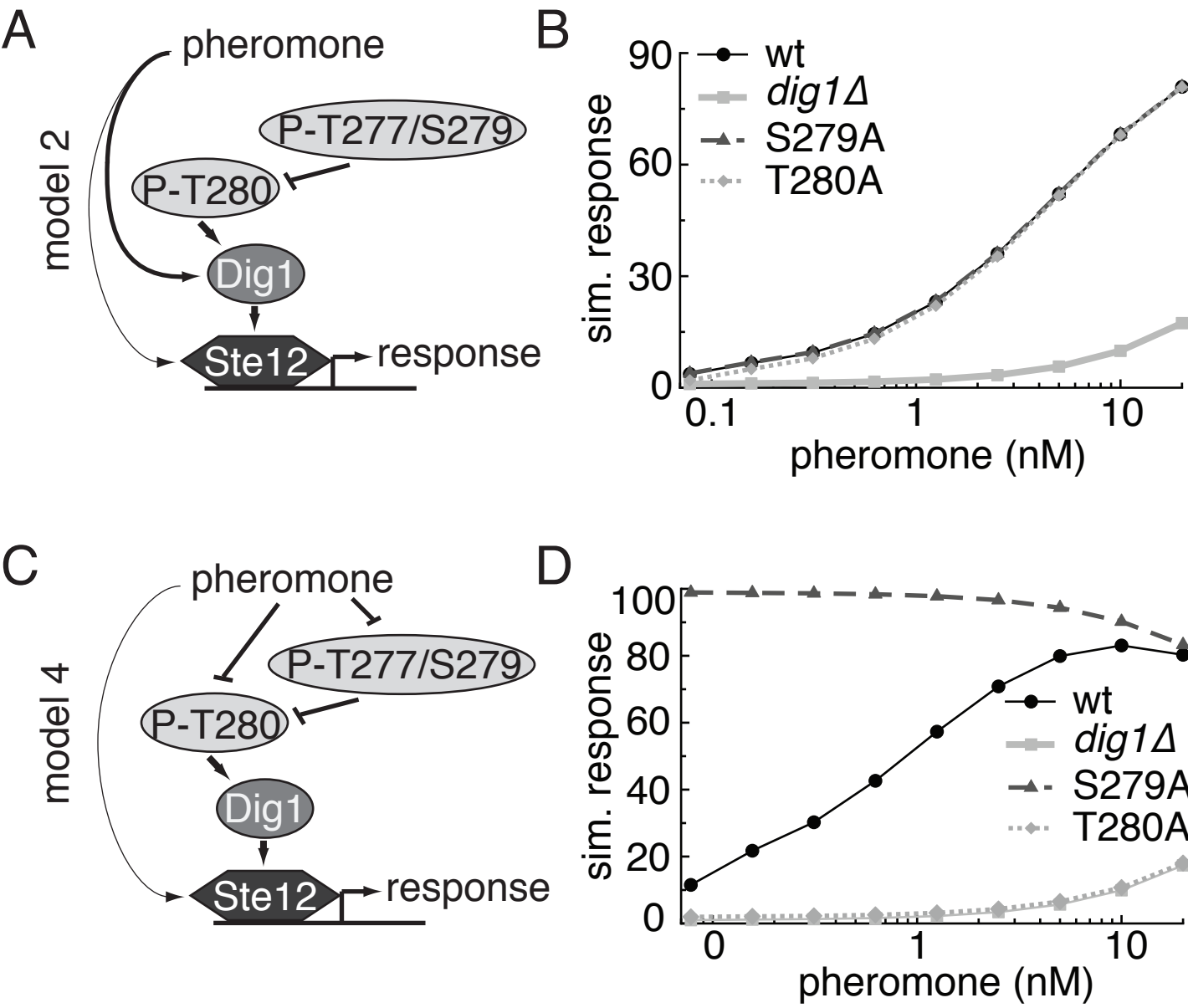

Supplement: Figure S8 — Dig1 model 2 and model 4 do not fit the experimental data. A. Cartoon depiction of Dig1 model 2, in which phosphorylation of T277 and T280 is constitutive and independent of pheromone. In this model, in order to have a dose dependent increase in Dig1 activity in the presence of constitutive phosphorylation of T277 and T280, we inserted a pheromone bypass directly on Dig1. B. Simulation of Dig1 model 2: To agree with the wild type response, the strength of this bypass was such that the contribution of phospho-T277 and -280 were negligible. Thus the mutants are identical to the wild type. C. Cartoon depiction of Dig1 model 4, in which dephosphorylation of both T277 and T280 is pheromone-dependent. D. Simulation of Dig1 model 4: The strength of the influence of each phosphorylation site had to be so strong, such that if one site was mutated, the effect of the other dominated over the pheromone response. Thus, the mutants cannot recapitulate the experimental dose responses. (PDF) [file pone.0056544.s008.pdf]

Figure S9

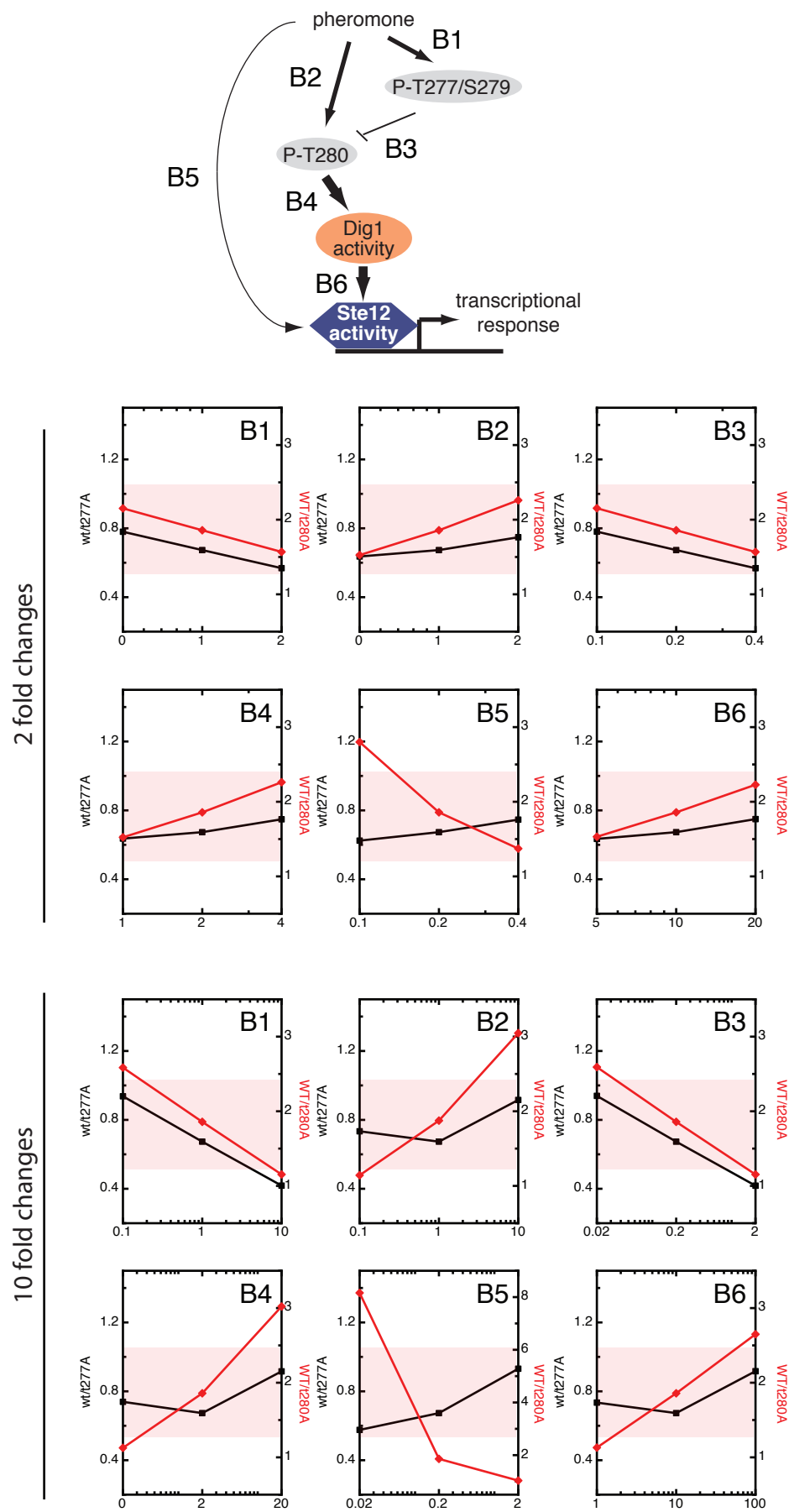

Supplement: Figure S9 — Dig1 model 1 is robust to 2-fold changes in parameters that determine the influence of the phosphorylation sites. The strength of each parameter depicted in the cartoon model were increased and decreased by 2-fold (top panels) and 10-fold (bottom panels), and the ratios of simulated output of wild type to T277A (black) and wild type to T280A (red) at 20 nM pheromone are plotted. The T277A phenotype persists (within pink boxes) with 2-fold changes to every parameter. The T280A phenotype persists except when the strength of the the Dig1-independent pheromone bypass (B4) becomes too strong. 10-fold changes to any parameter significantly change the simulation results. (PDF) [file pone.0056544.s009.pdf]

Figure S10

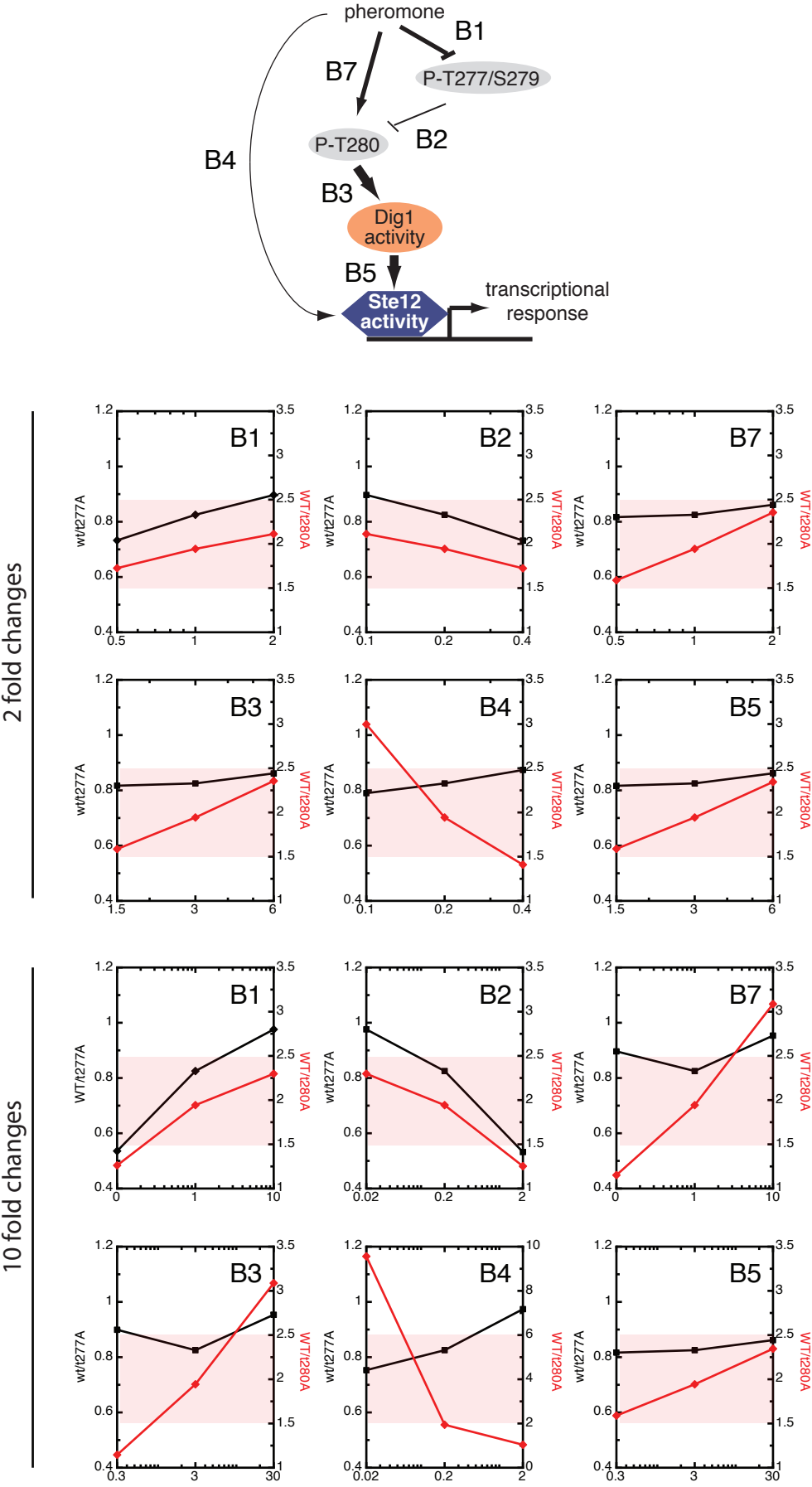

Supplement: Figure S10 — Dig1 model 3 is robust to 2-fold changes in parameters that determine the influence of the phosphorylation sites. The strength of each parameter depicted in the cartoon model were increased and decreased by 2-fold (top panels) and 10-fold (bottom panels), and the ratios of simulated output of wild type to T277A (black) and wild type to T280A (red) at 20 nM pheromone are plotted. The T277A phenotype persists (within pink boxes) with 2-fold changes to every parameter. The T280A phenotype persists except when the strength of the the Dig1-independent pheromone bypass (B5) becomes too strong. 10-fold changes to any parameter significantly change the simulation results. (PDF) [file pone.0056544.s010.pdf]
