# Supplementary material for: Assigning Quantitative Function to Post-Translational Modifications Reveals Multiple Sites of Phosphorylation That Tune Yeast Pheromone Signaling Output
Source: PLoS One. 2013 Mar 12;8(3):e56544. doi: 10.1371/journal.pone.0056544 (PMC3595240; doi:10.1371/journal.pone.0056544)
Supplement: Figure S7 — Dig1S269A,S272A,S275A activates the pheromone response like wild type. A. Alignment of the full tryptic peptide identified by mass spec as being phosphorylated against Dig1 orthologs in other yeast species. B. Cells bearing Dig1, Dig1S269A,S272A,S275A , Dig1T277A,S279A,T280 and cells deleted for DIG1 treated with 20nM pheromone for 3hrs. Dig1S269A,S272A,S275A is indistinguishable from wild type. (PDF) [file pone.0056544.s007.pdf]

Figure S7

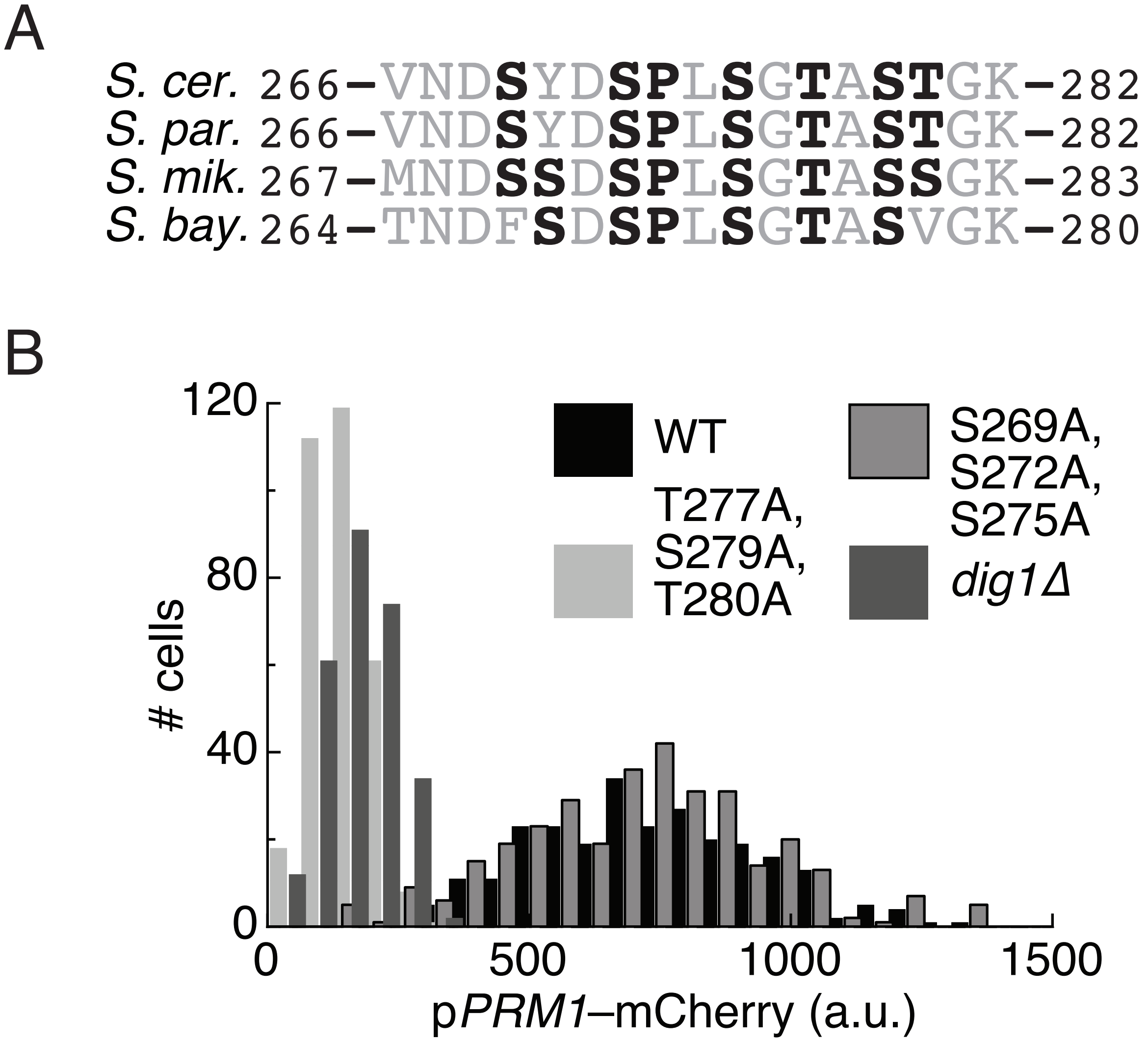

A
S. cer. 266–VNDSYDSPLSGTASTGK–282
S. par. 266–VNDSYDSPLSGTASTGK–282
S. mik. 267–MNDSSDSPLSGTASSGK–283
S. bay. 264–TNDFSDSPLSGTASVGK–280
B
120
80
40
0
cells
0
500
1000
1500
pPRM1–mCherry (a.u.)
WT
T277A, S279A, T280A
S269A, S272A, S275A
dig1Δ
