# Supplementary material for: Assigning Quantitative Function to Post-Translational Modifications Reveals Multiple Sites of Phosphorylation That Tune Yeast Pheromone Signaling Output
Source: PLoS One. 2013 Mar 12;8(3):e56544. doi: 10.1371/journal.pone.0056544 (PMC3595240; doi:10.1371/journal.pone.0056544)
Supplement: Table S1 — Strains used in this study. (DOCX) [file pone.0056544.s011.docx]

| TABLE S1: Strains used in this study | | |
| --- | --- | --- |
| Yeast Strains | Background | Relevant Genotype |
| ACLY379 | W303a | MATa bar1Δ |
| ACLY379pch | W303a | MATa bar1Δ Δprm1::P_prm1_-mCHERRY::hphMX6 |
| TCY3154 | W303a | MATa bar1Δ cdc28-as2 Δprm1::P_PRM1_-YFP::his5^+^  trp1::pACT1-CFP::TRP1 |
| RCY1130pch | W303a | MATa bar1Δ Δdig1::DIG1-YFP::URA3  Δste12::CFP-STE12::ura3 Δprm1::P_prm1_-mCHERRY::hphMX6 |
| DPY112 | W303a | MATa bar1Δ Δste12::kanMX4 Δprm1::P_prm1_-mCHERRY::hphMX6 |
| DPY250 | W303a | MATa bar1Δ Δste50::hphMX6 cdc28-as2 Δprm1::P_PRM1_-YFP::his5^+^  trp1::pACT1-CFP::TRP1 |
| RCY2005pch | W303a | MATa bar1Δ Δdig1::ura3 Δste12::CFP-STE12::ura3  Δprm1::P_prm1_-mCHERRY::hphMX6 |
| DPY1203 | W303a | MATa bar1Δ Δste12::ste12^S400A,S402A,T405A,S406A^ ::URA3  Δprm1::Pprm1-mCHERRY::hphMX6 |
| CRY1004 | W303a | MATa bar1, ste12::ste12^S400A^::URA3, prm1::Pprm1-mCHERRY::hphMX6 |
| CRY1005 | W303a | MATa bar1, ste12::ste12^S402A^::URA3, prm1::Pprm1-mCHERRY::hphMX6 |
| CRY1006 | W303a | MATa bar1, ste12::ste12^T405A^::URA3, prm1::Pprm1-mCHERRY::hphMX6 |
| CRY1007 | W303a | MATa bar1, ste12::ste12^S406A^::URA3, prm1::Pprm1-mCHERRY::hphMX6 |
| DPY1204 | W303a | MATa bar1Δ Δste12::ste12^T525A::URA3^::URA3  Δprm1::Pprm1-mCHERRY::hphMX6 |
| DPY1001 | W303a | MATa bar1Δ Δdig1::dig1^S126A,S127A,S129A^-YFP::URA3  Δste12::CFP-STE12::ura3 Δprm1::Pprm1-mCHERRY::hphMX6 |
| DPY1003 | W303a | MATa bar1Δ Δdig1::dig1^T277A,S279A,T280A^-YFP::URA3  Δste12::CFP-STE12::ura3 Δprm1::Pprm1-mCHERRY::hphMX6 |
| TCY3328 | W303a | MATa bar1Δ Δdig1::dig1^T277A^-YFP::URA3  Δste12::CFP-STE12::ura3 Δprm1::Pprm1-mCHERRY::hphMX6 |
| TCY3329 | W303a | MATa bar1Δ Δdig1::dig1^S279A^-YFP::URA3  Δste12::CFP-STE12::ura3 Δprm1::Pprm1-mCHERRY::hphMX6 |
| TCY3330 | W303a | MATa bar1Δ Δdig1::dig1^T280A^-YFP::URA3  Δste12::CFP-STE12::ura3 Δprm1::Pprm1-mCHERRY::hphMX6 |
| DPY5001 | W303a | MATa bar1Δ Δste50::ste50^S202A,T205A^::URA3 cdc28-as2  Δprm1::PPRM1-YFP-his5+ trp1::pACT1-CFP::TRP1 |
| TCY3344.2 | W303a | MATa bar1Δ Δste50::ste50^S202A^::URA3 cdc28-as2  Δprm1::PPRM1-YFP-his5+ trp1::pACT1-CFP::TRP1 |
| TCY3345.2 | W303a | MATa bar1Δ Δste50::ste50^T205A^::URA3 cdc28-as2  Δprm1::PPRM1-YFP-his5+ trp1::pACT1-CFP::TRP1 |
| *E. Coli* Strains | | Commercial Source |
| BL21CodonPlus(DE3) | | Stratagene, Inc. (now Agilent Technologies, Santa Clara, CA) |
| XL1-Blue Supercompetent Cells |  | Stratagene, Inc. (now Agilent Technologies, Santa Clara, CA) |
| XL1-Blue Electroporation-Competent Cells |  | Stratagene, Inc. (now Agilent Technologies, Santa Clara, CA) |
| DH5α |  | Invitrogen, Inc (Carlsbad, CA) |
