# Supplementary material for: Assigning Quantitative Function to Post-Translational Modifications Reveals Multiple Sites of Phosphorylation That Tune Yeast Pheromone Signaling Output
Source: PLoS One. 2013 Mar 12;8(3):e56544. doi: 10.1371/journal.pone.0056544 (PMC3595240; doi:10.1371/journal.pone.0056544)
Supplement: Table S2 — Primers used for strain construction and mutagenesis. (DOCX) [file pone.0056544.s012.docx]

| TABLE S2: Primers used for strain construction and mutagenesis | |
| --- | --- |
| Primer name | Primer sequence |
| STE12KOf | 5’-ggaaccgctttctttatttgaattgtcttgttcaccaagg  CGGATCCCCGGGTTAATTAA-3’ |
| STE12KOr | 5’-tttttaattcttgtatcataaattcaaaaattatattata  TCGATGAATTCGAGCTCGTT-3’ |
| STE50KOf | 5'-gtactagcagagatagcaaatcagatggaggacggtaaacaggc CGGATCCCCGGGTTAATTAA-3' |
| STE50KOr | 5'-tacacttaaaccaccatcctcgtcatcatattcaacatatctca TCGATGAATTCGAGCTCGTT-3' |
| STE12M3f | 5'-ataacaaggaaaaattggtaGctcctGCcgaccctGccGCctacatgaag-3' |
| STE12M3r | 5'-taccaatttttccttgttattgttagatg-3' |
| STE12-s400a-f | 5'-ataacaaggaaaaattggtaGcGcctagcgacc-3' |
| STE12-s400a-r | 5'-taccaatttttccttgttattgttagatga-3' |
| STE12-s402a-f | 5'-aggaaaaattggtatctcctGCcgaccctacc-3' |
| STE12-s402a-r | 5'-aggagataccaatttttccttgttattgtt-3' |
| STE12-t405a-f | 5'-tggtatctcctagcgaccctGcTagctacatga-3' |
| STE12-t405a-r | 5'-agggtcgctaggagataccaatttttcctt-3' |
| STE12-s406a-f | 5'-tatctcctagcgaccctaccGCctacatgaag-3' |
| STE12-s406a-r | 5'-ggtagggtcgctaggagataccaatttttc-3' |
| STE12M4f | 5'-acctatgttgggagaccatacGcaccgaattatagGtcgacaccag-3' |
| STE12M4r | 5'-gtatggtctcccaacataggttggagctgg-3' |
| DIG1M1f | 5'-ttaaggacggcaacttggctGcAGctaacGctgcacattttcc-3' |
| DIG1M1r | 5'-agccaagttgccgtccttaatattaccaccgtg-3' |
| DIG1M3f | 5'-atgattcgcctttgagtggcGccgctGccGctgggaaaac-3' |
| DIG1M3r | 5'-gccactcaaaggcgaatcatatgagtcgtt-3' |
| DIG1-t277a-f | 5’-AtgattcgcctttgagtggcGccgcttccactgggaaaac-3’ |
| DIG1-t277a-r | 5’-gccactcaaaggcgaatcatatgagtcgtt-3’ |
| DIG1-s279a-f | 5’-cgcctttgagtggcaccgctGCCactggCaaaaccCGCcgatccgagg-3’ |
| DIG1-s279a-r | 5’-agcggtgccactcaaaggcgaatcatatga-3’ |
| DIG1-t280a-f | 5’-ctttgagtggcaccgcttccGctgggaaaac-3’ |
| DIG1-t280a-r | 5’-ggaagcggtgccactcaaaggcgaatcata-3’ |
| STE50M1f | 5'-aatcaccgtcaaggagagaaGccccggtaGcggtatttagg-3' |
| STE50M1r | 5'-ttctctccttgacggtgattgtcggttatg-3' |
| STE50-s205a-f | 5'-caaggagagaaGCCccggtaACGgtatttag-3' |
| STE50-s205a-r | 5'-taccggggcttctctccttgacggtgattg-3' |
| STE50-t202a-f | 5'-aatcaccgtcaaggagagaaTCCccggtaGC-3' |
| STE50-t202a-r | 5'-ttctctccttgacggtgattgtcggttatg-3' |
