# Supplementary material for: Assigning Quantitative Function to Post-Translational Modifications Reveals Multiple Sites of Phosphorylation That Tune Yeast Pheromone Signaling Output
Source: PLoS One. 2013 Mar 12;8(3):e56544. doi: 10.1371/journal.pone.0056544 (PMC3595240; doi:10.1371/journal.pone.0056544)
Supplement: Table S3 — Plasmids used in this study. (DOCX) [file pone.0056544.s013.docx]

| TABLE S3: Plasmids used or created in this study | |
| --- | --- |
| Plasmid Name | Product of Insert |
| pRS406 | no insert |
| pSTE12-406 | Ste12 |
| pSTE12m3-406 | Ste12^S400A,S402A,T405A,S406A^ |
| pSTE12s400a-406 | Ste12^S400A^ |
| pSTE12s402a-406 | Ste12^S402A^ |
| pSTE12t405a-406 | Ste12^T405A^ |
| pSTE12s406a-406 | Ste12^S406A^ |
| pSTE12m4-406 | Ste12^T525A^ |
| pDIG1YFP-406 | Dig1-YFP |
| pDIG1m1YFP-406 | Dig1^S126A,S127A,S129A^-YFP |
| pDIG1m3YFP-406 | Dig1^T277A,S279A,T280A^-YFP |
| pDIG1t277aYFP-406 | Dig1^T277A^-YFP |
| pDIG1s279aYFP-406 | Dig1^S279A^-YFP |
| pDIG1t280aYFP-406 | Dig1^T280A^-YFP |
| pSTE50-406 | Ste50 |
| pSTE50m1-406 | Ste50^S202A,T205A^ |
| pSTE50s202a-406 | Ste50^S202A^ |
| pSTE50t205a-406 | Ste50^T205A^ |
