# Supplementary material for: Assigning Quantitative Function to Post-Translational Modifications Reveals Multiple Sites of Phosphorylation That Tune Yeast Pheromone Signaling Output
Source: PLoS One. 2013 Mar 12;8(3):e56544. doi: 10.1371/journal.pone.0056544 (PMC3595240; doi:10.1371/journal.pone.0056544)
Supplement: Table S4 — Public database links to phosphopeptides analyzed in this study. (DOCX) [file pone.0056544.s014.docx]

| Table S4: Links to Data on Phosphorylated Peptides Present in Public Databases | | | | |
| --- | --- | --- | --- | --- |
| Protein | Peptide | URL for phosphogrid database citation | URL for phosphopep database citation | URL for phosida database citation |
| Ste12 | L398-K409 | http://www.phosphogrid.org/sites/36519/YHR084W.phospho?topSearch=Ste12 | Search term: YHR084W  http://www.sbeams.org/devDC/sbeams/cgi/Glycopeptide//peptideSearch.cgi | Search term: Ste12  http://www.phosida.com/ |
| Dig1 | V266-K282 | http://www.phosphogrid.org/sites/36131/YPL049C.phospho?topSearch=Dig1 | Search term: YPL049C  http://www.sbeams.org/devDC/sbeams/cgi/Glycopeptide//peptideSearch.cgi | Search term: Dig1  http://www.phosida.com/ |
| Ste50 | R200-R208 | http://www.phosphogrid.org/sites/30951/YCL032W.phospho?topSearch=Ste50 | Search term: YCL032W <http://www.sbeams.org/devDC/sbeams/cgi/Glycopeptide//peptideSearch.cgi> | Search term: Ste50  http://www.phosida.com/ |
