# Supplementary material for: Assigning Quantitative Function to Post-Translational Modifications Reveals Multiple Sites of Phosphorylation That Tune Yeast Pheromone Signaling Output
Source: PLoS One. 2013 Mar 12;8(3):e56544. doi: 10.1371/journal.pone.0056544 (PMC3595240; doi:10.1371/journal.pone.0056544)
Supplement: Table S5 — P-values for mutant phenotypes. (DOCX) [file pone.0056544.s015.docx]

Table S5: p values for mutants vs. wild type

| Ste12 | *p* value |
| --- | --- |
| wt | 1 |
| *ste12∆* | 0 |
| 4x mutant | 1.17E-102 |
| S400A | 5.23E-101 |
| S402A | 1.11E-08 |
| T405A | 0.32294 |
| S406A | 3.73E-03 |
| T525A | 1.44E-72 |
|  |  |
| Dig1 | *p* value |
| wt | 1 |
| *dig1∆* | 5.66E-86 |
| 3x mutant | 2.34E-104 |
| T277A | 2.12E-45 |
| S279A | 1.11E-52 |
| T280A | 9.45E-79 |
|  |  |
| Ste50 | *p* value |
| wt | 1 |
| *ste50∆* | 1.01836E-82 |
| 2x mutant | 1.59092E-06 |
| T202A | 3.98557E-08 |
| S205A | 0.35830952 |
