# Supplementary material for: Assigning Quantitative Function to Post-Translational Modifications Reveals Multiple Sites of Phosphorylation That Tune Yeast Pheromone Signaling Output
Source: PLoS One. 2013 Mar 12;8(3):e56544. doi: 10.1371/journal.pone.0056544 (PMC3595240; doi:10.1371/journal.pone.0056544)
Supplement: Materials and Methods S1 — (DOCX) [file pone.0056544.s016.docx]

MATERIALS AND METHODS

1.0 CELL GROWTH AND REAGENTS

Routine growth of yeast strains was performed as described (*1*). Strains were grown on YAPD plates (YPD with supplemental adenine) and/or YAPD liquid (YPD with supplemental adenine) media. Yeast deletions were selected on YAPD/HygB or YAPD/Kan plates. Mutant strains were grown on SD plates and/or SD liquid media (yeast nitrogen base, 2% glucose, with appropriate selection for auxotrophic markers).

1.1 Pheromone synthesis

Alpha factor (or pheromone; Trp-His-Trp-Leu-Gln-Leu-Lys-Pro-Gly-Gln-Pro-Met-Tyr) was ordered from and synthesized at the W.M. Keck Foundation Biotechnology Resource Laboratory (Yale University). We made a 1 mM stock solution and stored it in aliquots at -80^o^C.

1.2 Pheromone treatment

For large-scale cell growth cells were treated with 1 μM pheromone for the time indicated. For both microscopic and flow cytometric studies cells were treated with 1-20 nM pheromone as indicated for the described time.

1.2 Reference assays

1.2.a Halo assays:

We qualitatively tested the pheromone response of all reference strains, deletion strains and phosphorylation site mutants by halo assay. We grew 4 mL overnight cultures to saturation in SD with appropriate auxotrophic selection (deletion strains and phosphorylation site mutants). We diluted overnights to OD_600_=1.0 in the same media, plated 100 μL (~10^6^ cells) on YAPD plates and spread cells on plates with glass beads. We pipeted 3 μL of 1 mM pheromone onto paper discs in the center of the plates and incubated the plates at 30°C for 20 hrs, following which the size of the halo of each strain was recorded.

1.2.b Pheromone sensitivity assays:

We qualitatively tested the pheromone sensitivity of all reference strains, deletion strains and phosphorylation site mutants. We prepared YAPD plates with 10-fold dilutions of pheromone, ranging from 1μM to 1nM. We diluted saturated overnight cultures of strains to OD_600_=1.0. On each of the four plates we spotted 10, 100 and 1000 cells of each strain and incubated the plates 30°C for 20 hrs, following which the growth of cells was recorded.

1.3 Reagents - Antibodies

1.3.a Custom rabbit polyclonal antibodies against pheromone pathway proteins:

We used custom rabbit polyclonal antibodies against pheromone pathway proteins (*2*).

1.3.b Commercial primary antibodies:

We monitored phosphorylation of the MAPKs (Fus3 and Kss1) in populations of cells by Western blot analysis with rabbit anti-phospho-p44/42 antibodies (Cell Signaling Technologies, Beverly, MA). We monitored the appearance of fluorescent protein fusion proteins by Western blot analysis using mouse monoclonal anti-GFP antibodies (JL-8) (BD Biosciences, Palo Alto, CA). We verified the relative abundance of cell extract per gel lane by Western blot analysis using a mouse monoclonal antibody against the reference protein, GAPDH (Abcam, Cambridge MA).

1.3.c Commercial secondary antibodies:

We used one of two fluorescently-labeled secondary antibodies to visualize all results on Westerns (Alexa Fluor^®^ 680 series, Invitrogen Co., Carlsbad, CA; IRDye^®^ 800 series, Rockland Immunochemicals Inc., Gilbertsville, PA).

2.0 STRAIN CONSTRUCTION

2.1 Construction of deletion strains

We created yeast strains lacking the ORFs *STE12*, *DIG1* and *STE50*. STE12: We deleted *STE12* from ACLY379 using PCR-mediated one-step replacement (*1*) with the pFA6a-kanMX6 template (*3*) and primer pair STE12KOf/STE12KOr, creating D12. We verified the deletion by PCR and Western blot analysis. Subsequently, we replaced the *PRM1* ORF in D12 with a PCR product containing the *mCHERRY* coding sequence, the *ADH1* terminator and the *hph* gene from pAG32-hphMX6 (*4*), creating DPY112. DIG1: We deleted *DIG1* by one-step excision of *ura3*-marked *DIG1-YFP* from RCY1130 using 5-FOA (*1*) to select for excision products, creating RCY2005. We verified the deletion using epifluorescent microscopy and Western blot analysis. Subsequently, we replaced the *PRM1* ORF in RCY2005 with a PCR product containing the *mCHERRY* coding sequence, the *ADH1* terminator and the *hph* gene from pAG32-hphMX6 (*4*) creating RCY2005pch. STE50: We deleted *STE50* from TCY3154 using PCR-mediated one-step replacement (*1*) with the pAG32-hphMX6 (*4*) template and primer pair STE50KOf/STE50KOr, creating DPY250. We verified the deletion by PCR and Western blot analysis.

2.2 Construction of strains containing mutant proteins

We created yeast strains with mutant alleles of *STE12*, *DIG1-YFP* and *STE50*.

2.2.a STE12 (Ste12^S400A,S402A,T405A,S406A^, Ste12^S400A^, Ste12^S402A^, Ste12^T405A^, Ste12^S406A^, and Ste12^T525A^):

To make the mutant *STE12* strains, we amplified the wild type STE12 gene (including 968 bp of endogenous promoter, the ORF and 634 bp of endogenous terminator) from W303a genomic DNA and cloned this PCR product into pRS406 at XhoI and EcoRI sites, creating pSTE12-406. We performed site-directed mutagenesis with the GeneTailor™ site directed mutagenesis kit (Invitrogen Co., Carlsbad, CA) using the following primer pairs: STE12M3f/STE12M3r, STE12-s400a-f/STE12-s400a-r, STE12-s402a-f/STE12-s402a-r, STE12-t405a-f/STE12-t405a-r, STE12-s406a-f/STE12-s406a-r and STE12M4f/STE12M4r and methylated pSTE12-406 as the template, creating pSTE12m3-406, pSTE12s400a-406, pSTE12s402a-406, pSTE12t405a-406, pSTE12s406a-406, and pSTE12m4-406. The sequences of the mutated plasmids were verified (MWG Biotech, High Point, NC). We linearized the mutant plasmids in the STE12 promoter region with BsiWI (New England Biolabs, Beverly, MA), and individually transformed them into DPY112 and plated on SD plates lacking uracil, creating DPY1203, CRY1004, CRY1005, CRY1006, CRY1007 and DPY1204. We verified the presence of and normal abundance of Ste12^S400A,S402A,T405A,S406A^, Ste12^S400A^, Ste12^S402A^, Ste12^T405A^, Ste12^S406A^, and Ste12^T525A^ proteins using Western blot analysis and the absence of the Ste12 protein in the Ste12D reference strain.

2.2.b DIG1 (Dig1^S126A,S127A,S129A^-YFP, Dig1^T277A,S279A,T280A^-YFP, Dig1^T277A^-YFP, Dig1^S279A^-YFP, and Dig1^T280A^-YFP):

To make the *DIG1-YFP* mutant strains, we performed site-directed mutagenesis and sent for sequencing as above, using primer pairs DIG1M1f/DIG1M1r, DIG1M3f/DIG1M3r, Dig1-t277a-f/Dig1-t277a-r, Dig1-s279a-f/Dig1-s279a-r, and Dig1-t280a-f/Dig1-t280a-r and methylated pDIG1YFP-406 (*3*) template, creating pDIG1m1YFP-406, pDIG1m3YFP-406, pDIG1t277aYFP-406, pDIG1s279aYFP-406, and pDIG1t280aYFP-406. We linearized the mutant plasmids in the *DIG1* promoter region with BstEII (New England Biolabs, Beverly, MA), individually transformed them into RCY2005pch and plated on SD plates lacking uracil, creating DPY1001, DPY1003, TCY3328, TCY3329, and TCY3330. We verified both the presence and normal abundance of Dig1^S126A,S127A,S129A^, Dig1^T277A,S279A,T280A^-YFP, Dig1^T277A^-YFP, Dig1^S279A^-YFP, and Dig1^T280A^-YFP proteins by Western blot analysis and the absence of the Dig1 protein in the Dig1D reference strain. We additionally verified that the Dig1-YFP proteins localized to the nucleus using epifluorescent microscopy.

2.2.c STE50 (Ste50^S202A,T205A^, Ste50^S202A^, and Ste50^T205A^):

To make the *STE50* mutant strains, we amplified the wild type STE50 gene (including 76 bp of endogenous promoter, the ORF and 384 bp of endogenous terminator) from W303 genomic DNA and cloned the PCR product into pRS406 at the XhoI site, creating pSTE50-406. We performed site-directed mutagenesis and sent for sequencing as above, using primer pairs STE50M1f/STE50M1r, STE50-s202a-f/STE50-s202a-r, and STE50-t205a-f/STE50-t205a-r and methylated pSTE50-406 as the template, creating pSTE50m1-406, pSTE50s202a-406, and pSTE50t205a-406. We linearized the mutant plasmids in the STE50 promoter region with PshAI (New England Biolabs, Beverly, MA), transformed into DPY250 and plated on SD plates lacking tryptophan and uracil, creating DPY5001, TCY3344.2, and TCY3345.2. We verified the presence and normal abundance of Ste50^S202A,T205A^, Ste50^S202A^, and Ste50^T205A^ proteins by Western blot analysis and the absence of the Ste50 protein in the Ste50D reference strain.

3.0 SINGLE CELL TRANSCRIPTION ASSAYS OF PHOSPHORYLATION MUTANT STRAINS

3.1 Fluorescent transcription assays of phosphorylation mutant strains

3.1.a Fluorescent transcription assay:

We quantitatively tested the pheromone response of all strains by measuring FP fluorescence in single cells as a surrogate for transcriptional output from a pheromone responsive promoter (P_PRM1_-YFP). Starting from single colonies on YAPD plates, we inoculated 4 mL cultures in YAPD. We monitored growth by A_600_ and diluted into 4 mL SDC at the end of the day to obtain mid-log phase cultures in the morning. We diluted cultures to OD_600_=0.2 in YAPD and grew to OD_600_=0.8. We centrifuged 1.5 mL cells for 30 sec at 15,000 rpm, resuspended in 1.5mL SDC with 40 μg/mL casein (from DIG nucleic acid detection kit, Roche Diagnostics Corporation, Indianapolis, IN) and sonicated the cells to disperse clumps. We pipetted 500 μL of each strain into a 96 well deep well plate for the untreated (no pheromone) samples and added 50μL 50 μg/mL cycloheximide (Calbiochem (EMD), San Diego, CA). We pipetted 495 μL of each strain into the same 96 well deep well plate and added 5 μL of 100μM pheromone in SDC/casein. We incubated the plate at 30°C for 15 minutes with shaking (300 rpm) and stopped the experiment with 50 μL 50 μg/mL cycloheximide in SDC/casein (Calbiochem (EMD), San Diego, CA). We incubated an additional 2.5 hrs at 30°C, shaking at 300 rpm, to allow for fluorophore maturation. We sonicated the cells again, diluted 1:20 in SDC/casein/cycloheximide, pipetted 200 μL into 96 well glass bottom plates and let the cells settle for 10 minutes. We performed optical microscopic cytometry and image capture as described elsewhere (*5, 6*) and image analysis, and data processing using the open source software packages Cell-ID and PAW (*7, 8*).

3.1.b STE12 (Ste12^S400A,S402A,T405A,S406A^, Ste12^S400A^, Ste12^S402A^, Ste12^T405A^, Ste12^S406A^ and Ste12^T525A^):

We compared pathway output in ACLY379pch, DPY112, DPY1203, CRY1004, CRY1005, CRY1006, and CRY1007. Starting from single colonies, we inoculated 4 mL cultures in SDC (ACLY379pch and DPY112) or SD/-U (DPY1203, CRY1004, CRY1005, CRY1006, and CRY1007) and grew over the course of the day to obtain log phase cells. We diluted overnight 4 mL cultures in the same media to obtain mid-log phase cells in the morning, at which point we adjusted to OD_600_=0.25 and allowed the cells to grow for one generation (90 minutes). We prepared a 4-fold dilution series of pheromone in SDC/casein ranging from 20 nM - 0.31nM. We aliquoted 7 x 0.5 mL of each dilution into a 96-well deep well plate, and to another set of 7 wells we added 0.5 mL SDC/casein/cycloheximide in order to measure baseline pathway output. We sonicated 1.0 mL of each culture to disperse cell clumps and added 50 μL of each culture to the wells containing SDC/casein/cycloheximide and the pheromone-dilution series. We incubated the plate at 30°C for 3 hours, shaking at 300rpm, at which point we stopped the experiment and allowed the fluorophores to mature as described above. We sonicated the plate and quantified reporter fluorescence by microscopy (3.1.a) and flow cytometry. 5 ul of cells was subjected to flow cytometric analysis using a BD LSR-II flow cytometer (UCSF QB3 core facility) equipped with a high throughput sampler, a 488 nm 100 mW laser, FITC emission filter and FACS DIVA software to compile .fcs files.

3.1.c DIG1 (Dig1^T277A,S279A,T280A^-YFP, Dig1^S277A^-YFP, Dig1^T279A^-YFP, and Dig1^S280A^-YFP):

We compared pathway output in RCY1130pch, RCY2005pch, DPY1003, TCY3328, TCY3329, and TCY3330. Starting from single colonies, we inoculated 4 mL cultures in SDC (RCY1130pch), SD/-W (RCY2005pch), or SD/-U (DPY1003, TCY3328, TCY3329, and TCY3330) and grew over the course of the day to obtain log phase cells. We diluted overnight 4 mL cultures in the same media to obtain mid-log phase cells in the morning, at which point we adjusted to OD_600_=0.25 and allowed the cells to grow for one generation (90 minutes). We prepared a 4-fold dilution series of pheromone in SDC/casein ranging from 20nM-0.31nM. We aliquoted 6 x 0.5mL of each dilution into a 96-well deep well plate, and to another set of 6 wells we added 0.5 mL SDC/casein/cycloheximide in order to measure baseline pathway output. We sonicated 1.0 mL of each culture to disperse cell clumps and added 50 μL of each culture to the wells containing SDC/casein/cycloheximide and the pheromone-dilution series. We incubated the plate at 30°C for 3 hours, shaking at 300 rpm, at which point we stopped the experiment and allowed the fluorophores to mature as described above. We sonicated the plate and quantified reporter fluorescence by microscopy (3.1.a).

3.1.d STE50 (Ste50^S202A,T205A^, Ste50^S202A^ and Ste50^T205A^):

We compared pathway output in TCY3154, DPY250, DPY5001, TCY3344.2, and TCY3345.2. Starting from single colonies, we inoculated 4 mL cultures in SD/-W (TYC3154 and DPY250), SD/-W-U (DPY5001, TCY3344.2, and TCY3345.2) and grew over the course of the day to obtain log phase cells. We diluted overnight 4 mL cultures in the same media to obtain mid-log phase cells in the morning, at which point we adjusted to OD_600_=0.25 and allowed the cells to grow for one generation (90 minutes). We prepared a 4-fold dilution series of pheromone in SDC/casein ranging from 20nM-0.31nM. We aliquoted 5 x 0.5 mL of each dilution into a 96-well deep well plate, and to another set of 5 wells we added 0.5 mL SDC/casein/cycloheximide in order to measure baseline pathway output. We sonicated 1.0 mL of each culture to disperse cell clumps and added 50μL of each culture to the wells containing SDC/casein/cycloheximide and the pheromone-dilution series. We incubated the plate at 30°C for 3 hours, shaking at 300 rpm, at which point we stopped the experiment and allowed the fluorophores to mature as described above. We sonicated the plate and quantified reporter fluorescence by microscopy (3.1.a).

4.0 BIOINFORMATIC ANALYSES

Previously determined sites of phosphorylation, protein domains, protein/protein interaction information and gene and protein sequences were obtained from primary literature, the Biobase YPD database [www.proteome.com] and the *Saccharomyces* genome database (S288C strain background) [www.yeastgenome.org].

We used tree-assisted ortholog alignments to identify *S. cerevisiae* pheromone pathway protein orthologs in other yeast species. In two cases (Fus3 and Kss1) orthologs from some species were not included in the ortholog assignments. In these cases we obtained the absent ortholog sequences from the *Y. lipolytica* database (*9*) from orthlogues assigned in (*10*) and from the Wapinski update (January 2009) that is available on SGD (yeastgenome.org) in the fungal orthogroups repository. We used the Yeast Gene Order Browser (YGOB) to distinguish orthologs and paralogs in cases of retained duplicates (*11*).

We aligned regions of *S. cerevisiae* Ste12, Dig1, and Ste50 to the best matching sequences in the orthologs identified by Wapinski et al. to demonstrate the conservation of motifs and phosphorylation sites.

5.0 MODELING WORK

For each protein of interest (Ste12, Dig1, Ste50), we wrote a set of ordinary differential equations (ODEs) to describe roles for phosphorylation sites in the context of pheromone signaling. All reaction equations are based on mass action kinetics, and a Hill function describes the transcriptional induction step. Although the models employ the standard mathematical approach of ODE-based mass action equations, the models qualitatively differ from standard approaches in two important ways.

First, instead of explicitly keeping track of concentrations of the species in physical units, the models represent the species in abstract, non-physical units of “activity”. These abstract species still exert their effects in proportion to their amounts, and thus still operate in a mass action paradigm. This method emphasizes the generic architectural constraints of the pathway and avoids the requirement of fitting specific unmeasured parameters.

Second, the models incorporate phosphorylation in a non-canonical way. Standard ODE models represent unmodified and modified proteins as distinct species that have their own production, decay and reaction rates. Here, in contrast, phosphorylation does not generate novel species with new properties, rather phosphorylation tunes the inherent activity of the native proteins by acting “in trans”. In this way, the models remain uncluttered by additional unmeasured parameters and assumptions, and therefore emphasize how the phosphorylation events tune the activity of the protein they modify. This approach could be used to study the role of any protein modification.

To implement the models, equations were coded and simulated across a pheromone dose response in MatLab R2008a using the ODE23s solver (code included as supplementary material). Parameters were all set to 1 to initiate the system, and were modified to fit the experimental data. Once a set of parameters was obtained that fit the experimental data, we performed sensitivity analysis by sweeping through 2- and 10-fold increases and decreases of each parameter and simulating the ratio of mutant to reference strain transcription in response to 20 nM pheromone (supplementary figures). The observed differences between the mutants and reference strain transcriptional responses were insensitive to most parameter changes. Sensitive parameters are discussed in the main text.
