# Supplementary material for: Assigning Quantitative Function to Post-Translational Modifications Reveals Multiple Sites of Phosphorylation That Tune Yeast Pheromone Signaling Output
Source: PLoS One. 2013 Mar 12;8(3):e56544. doi: 10.1371/journal.pone.0056544 (PMC3595240; doi:10.1371/journal.pone.0056544)
Supplement: Text S1 — Matlab code for simulations. (DOCX) [file pone.0056544.s017.docx]

**MATLAB CODE**

function Ste12_1

global parameter

% dose response loop

%for pheromone=[0,0.15625,0.3125,0.625,1.25,2.5,5,10,20]

for parameter=[5,10,20]

pheromone=20;

to=0;tf =25;tspan=[to tf];

%lk=500;

%axis(lk*[-20 20 -1000 1000]);

grid on;

hold on

% y1 is pheromone

% y2 is MAPK activity

% y3 is P-S400 activity

% y4 is P-T525 activity

% y5 is Ste12 activity

% y6 is transcriptional response

% initial conditions

yo(1)=pheromone;

yo(2)=0;

yo(3)=0;

yo(4)=0;

yo(5)=0;

yo(6)=0;

options = odeset('reltol',1e-3);

%[yo(1) yo(2) but] = ginput(1)

%while but ~= 3

grid on;

hold on

[t ,y] = ode23s('Ste12_1_eqn',tspan,yo,options);

y1=([1 0 0 0 0 0]*y')';

y2=([0 1 0 0 0 0]*y')';

y3=([0 0 1 0 0 0]*y')';

y4=([0 0 0 1 0 0]*y')';

y5=([0 0 0 0 1 0]*y')';

y6=([0 0 0 0 0 1]*y')';

%y2=([0 1]*y')';

% y3=([0 0 1]*y')';

%figure(2)

%plot(t,y3)

%hold on;

%figure(1);

%plot(y1,y2,'b');

%m=length(t);

%plot(y1(m),y2(m),'bo');

%[yo(1) yo(2) but] = ginput(1);

%if but==2

%yo(1)=y1(m);yo(2)=y2(m);

%end

%end

y6

figure(1)

plot(t,y6,'k')

%figure(2);

%plot(t,y2,'g');

% figure(3)

% plot(t,y3,'g');

% hold on;

% plot(t,y2,'b');

%end

end

end

-----------------------------------------------------------

function ty=Ste12_1_eqn(t,y)

global parameter

% paramaters by variable

% MAPK

alpha1=0.1;

beta1=1;

gamma1=1;

% P-S400

alpha2=0.1;

beta2=1;

gamma2=1;

% P-T525

alpha3=0.1;

beta3=1;

gamma3=1;

% Ste12

alpha4=0.1;

beta4=0.1;

beta5=1;

beta6=1;

gamma4=parameter;

% txn response

alpha5=0.1;

beta7=100;

gamma5=1;

n1=1;

k1=1;

% y1 is pheromone

% y2 is MAPK activity

% y3 is P-S400 activity

% y4 is P-T525 activity

% y5 is Ste12 activity

% y6 is transcriptional response

% Hill equation for transcriptional response

hillterm=((y(5)/k1)^n1)/(1+(y(5)/k1)^n1);

ty(1)=0;

ty(2)=alpha1-gamma1*y(2)+beta1*y(1);

ty(3)=alpha2-gamma2*y(3)+beta2*y(2);

ty(4)=alpha3-gamma3*y(4)+beta3*y(2);

ty(5)=alpha4-gamma4*y(5)+beta4*y(2)+beta5*y(3)+beta6*y(4);

ty(6)=alpha5-gamma5*y(6)+beta7*hillterm;

ty=[ty(1);ty(2);ty(3);ty(4);ty(5);ty(6)];

end

-----------------------------------------------------------

function Dig1_1

global parameter

% dose response loop

%for pheromone=[0,0.15625,0.3125,0.625,1.25,2.5,5,10,20]

for parameter=[1,10,100]

pheromone=20;

to=0;tf =25;tspan=[to tf];

%lk=500;

%axis(lk*[-20 20 -1000 1000]);

grid on;

hold on

% y1 is pheromone

% y2 is MAPK activity

% y3 is P-T280 activity

% y4 is P-T277 AND P-S279 activity

% y5 is Dig1 activity

% y6 is Ste12 activity

% y7 is transcriptional response

% initial conditions

yo(1)=pheromone;

yo(2)=0;

yo(3)=0;

yo(4)=0;

yo(5)=0;

yo(6)=0;

options = odeset('reltol',1e-3);

%[yo(1) yo(2) but] = ginput(1)

%while but ~= 3

grid on;

hold on

[t ,y] = ode23s('Dig1_1_eqn',tspan,yo,options);

y1=([1 0 0 0 0 0]*y')';

y2=([0 1 0 0 0 0]*y')';

y3=([0 0 1 0 0 0]*y')';

y4=([0 0 0 1 0 0]*y')';

y5=([0 0 0 0 1 0]*y')';

y6=([0 0 0 0 0 1]*y')';

%y2=([0 1]*y')';

% y3=([0 0 1]*y')';

%figure(2)

%plot(t,y3)

%hold on;

%figure(1);

%plot(y1,y2,'b');

%m=length(t);

%plot(y1(m),y2(m),'bo');

%[yo(1) yo(2) but] = ginput(1);

%if but==2

%yo(1)=y1(m);yo(2)=y2(m);

%end

%end

y6

figure(1)

%plot(t,y2,'g')

%plot(t,y3,'r')

%plot(t,y4,'k')

plot(t,y6,'b')

%figure(2);

%plot(t,y2,'g');

% figure(3)

% plot(t,y3,'g');

% hold on;

% plot(t,y2,'b');

end

end

-----------------------------------------------------------

function ty=Dig1_1_eqn(t,y)

global parameter

% paramaters by variable

% P-T277 AND P-S279

alpha1=0.1;

beta1=1;

gamma1=1;

% P-T280

alpha2=0.1;

beta2=1;

beta3=0.2;

gamma2=1;

% Dig1

alpha3=0.1;

beta4=2;

gamma3=10;

% Ste12

alpha4=0.1;

beta5=0.2;

beta6=parameter;

gamma4=10;

% txn response

alpha5=0.01;

beta7=100;

gamma5=1;

n1=1;

k1=1;

% y1 is pheromone

% y2 is P-T277 AND P-S279 activity

% y3 is P-T280 activity

% y4 is Dig1 activity

% y5 is Ste12 activity

% y6 is transcriptional response

% Hill equation for transcriptional response

hillterm=((y(5)/k1)^n1)/(1+(y(5)/k1)^n1);

ty(1)=0;

ty(2)=alpha1-gamma1*y(2)+beta1*y(1);

ty(3)=alpha2-gamma2*y(3)+beta2*y(1)-beta3*y(2)*y(3);

ty(4)=alpha3-gamma3*y(4)+beta4*y(3);

ty(5)=alpha4-gamma4*y(5)+beta5*y(1)+beta6*y(4);

ty(6)=alpha5-gamma5*y(6)+beta7*hillterm;

ty=[ty(1);ty(2);ty(3);ty(4);ty(5);ty(6)];

end

-----------------------------------------------------------

function Dig1_3

global parameter

% dose response loop

%for pheromone=[0,0.15625,0.3125,0.625,1.25,2.5,5,10,20]

for parameter=[0.02,0.2,2]

pheromone=20;

to=0;tf =25;tspan=[to tf];

%lk=500;

%axis(lk*[-20 20 -1000 1000]);

% y1 is pheromone

% y2 is P-T277 AND P-S279 activity

% y3 is P-T280 activity

% y4 is Dig1 activity

% y5 is Ste12 activity

% y6 is transcriptional response

% initial conditions

yo(1)=pheromone;

yo(2)=0;

yo(3)=0;

yo(4)=0;

yo(5)=0;

yo(6)=0;

options = odeset('reltol',1e-3);

%[yo(1) yo(2) but] = ginput(1)

%while but ~= 3

[t ,y] = ode23s('Dig1_3_eqn',tspan,yo,options);

y1=([1 0 0 0 0 0]*y')';

y2=([0 1 0 0 0 0]*y')';

y3=([0 0 1 0 0 0]*y')';

y4=([0 0 0 1 0 0]*y')';

y5=([0 0 0 0 1 0]*y')';

y6=([0 0 0 0 0 1]*y')';

%y2=([0 1]*y')';

% y3=([0 0 1]*y')';

%figure(2)

%plot(t,y3)

%hold on;

%figure(1);

%plot(y1,y2,'b');

%m=length(t);

%plot(y1(m),y2(m),'bo');

%[yo(1) yo(2) but] = ginput(1);

%if but==2

%yo(1)=y1(m);yo(2)=y2(m);

%end

%end

y6

grid on;

hold on

figure(1)

%plot(t,y3,'b')

%plot(t,y2,'r')

plot(t,y6,'b')

%figure(2);

%plot(t,y2,'g');

% figure(3)

% plot(t,y3,'g');

% hold on;

% plot(t,y2,'b');

end

end

-----------------------------------------------------------

function ty=Dig1_3_eqn(t,y)

global parameter

% paramaters by variable

% P-T277 AND P-S279

alpha1=100;

beta1=1;

gamma1=0.1;

% P-T280

alpha2=0.1;

beta2=0.2;

beta7=1;

gamma2=1;

% Dig1

alpha3=0.1;

beta3=3;

gamma3=10;

% Ste12

alpha4=0.1;

beta4=parameter;

beta5=3;

gamma4=10;

% txn response

alpha5=0.1;

beta6=100;

gamma5=1;

n1=1;

k1=1;

% y1 is pheromone

% y2 is P-T277 AND P-S279 activity

% y3 is P-T280 activity

% y4 is Dig1 activity

% y5 is Ste12 activity

% y6 is transcriptional response

% Hill equation for transcriptional response

hillterm=((y(5)/k1)^n1)/(1+(y(5)/k1)^n1);

ty(1)=0;

ty(2)=alpha1-gamma1*y(2)-beta1*y(1)*y(2);

ty(3)=alpha2-gamma2*y(3)-beta2*y(2)*y(3)+beta7*y(1);

ty(4)=alpha3-gamma3*y(4)+beta3*y(3);

ty(5)=alpha4-gamma4*y(5)+beta4*y(1)+beta5*y(4);

ty(6)=alpha5-gamma5*y(6)+beta6*hillterm;

ty=[ty(1);ty(2);ty(3);ty(4);ty(5);ty(6)];

end

-----------------------------------------------------------

function Ste50_1

global parameter

% dose response loop

%for pheromone=[0,0.15625,0.3125,0.625,1.25,2.5,5,10,20]

for parameter=[0.1,1,10]

pheromone=20;

to=0;tf =25;tspan=[to tf];

%lk=500;

%axis(lk*[-20 20 -1000 1000]);

grid on;

hold on

% y1 is pheromone

% y2 is P-S202

% y3 is Ste50

% y4 is MAPK

% y5 is transcriptional response

% initial conditions

yo(1)=pheromone;

yo(2)=0;

yo(3)=0;

yo(4)=0;

yo(5)=0;

options = odeset('reltol',1e-3);

%[yo(1) yo(2) but] = ginput(1)

%while but ~= 3

grid on;

hold on

[t ,y] = ode23s('Ste50_1_eqn',tspan,yo,options);

y1=([1 0 0 0 0]*y')';

y2=([0 1 0 0 0]*y')';

y3=([0 0 1 0 0]*y')';

y4=([0 0 0 1 0]*y')';

y5=([0 0 0 0 1]*y')';

%y2=([0 1]*y')';

% y3=([0 0 1]*y')';

%figure(2)

%plot(t,y3)

%hold on;

%figure(1);

%plot(y1,y2,'b');

%m=length(t);

%plot(y1(m),y2(m),'bo');

%[yo(1) yo(2) but] = ginput(1);

%if but==2

%yo(1)=y1(m);yo(2)=y2(m);

%end

%end

y5

figure(1)

plot(t,y5,'k')

%figure(2);

%plot(t,y2,'g');

% figure(3)

% plot(t,y3,'g');

% hold on;

% plot(t,y2,'b');

end

end

-----------------------------------------------------------

function ty=Ste50_1_eqn(t,y)

global parameter

% paramaters by variable

% P-S202

alpha0=0.1;

beta1=1;

gamma0=1;

% Ste50

alpha1=0.1;

beta2=1;

beta3=1;

gamma1=1;

% MAPK

alpha2=0.1;

beta4=parameter;

beta6=0.01;

gamma2=1;

% txn response

alpha3=0.1;

beta5=100;

gamma3=1;

n1=1;

k1=1;

% y1 is pheromone

% y2 is P-S202 activity

% y3 is Ste50 activity

% y4 is MAPK activity

% y5 is transcriptional response

% Hill equation for transcriptional response

hillterm=((y(4)/k1)^n1)/(1+(y(4)/k1)^n1);

ty(1)=0;

ty(2)=alpha0-gamma0*y(2)+beta1*y(4);

ty(3)=alpha1-gamma1*y(3)+beta2*y(1)-beta3*y(2)*y(3);

ty(4)=alpha2-gamma2*y(4)+beta4*y(3)+beta6*y(1);

ty(5)=alpha3-gamma3*y(5)+beta5*hillterm;

ty=[ty(1);ty(2);ty(3);ty(4);ty(5)];

end
